# Researchers' sex/gender identity influences how sex/gender question is investigated in neuroscience: an example from an OHBM meeting

**DOI:** 10.1007/s00429-023-02750-8

**Published:** 2024-02-16

**Authors:** Maria Picó-Pérez, Elena Abalos Marco, Lindsey T. Thurston, Valerie Ambrosi, Sarah Genon, Katherine L. Bryant, Ana Belén Martínez, Lu Ciccia, Anelis Kaiser Trujillo

**Affiliations:** 1 Life and Health Sciences Research Institute, School of Medicine, University of Minho, Braga, Portugal; 2 ICVS/3B’s, PT Government Associate Laboratory, Braga, Guimarães, Portugal; 3 Departamento de Psicología Básica, Clínica y Psicobiología, Universitat Jaume I, Castelló de la Plana, Spain; 4 Gender in STEM, University of Freiburg, Freiburg, Germany; 5 Department of Psychology, University of Toronto, Toronto, Canada; 6 Institute Technology and Education, University of Bremen, Bremen, Germany; 7 Institute of Neuroscience and Medicine, Research Centre Jülich, Jülich, Germany; 8 Laboratoire de Psychologie Cognitive, Université Aix-Marseille, Marseille, France; 9 Filosofía de la Biología, Universidad de Buenos Aires, Buenos Aires, Argentina; 10 Centro de Investigaciones y Estudios de Género, Universidad Nacional Autónoma de México, Ciudad de México, México

**Keywords:** Equality, Sex/gender research, OHBM, Neurofeminism

## Abstract

Gender inequality and diversity in STEM is a challenging field of research. Although the relation between the sex/gender of the researcher and the scientific research practices has been previously examined, less interest has been demonstrated towards the relation between sex/gender of the researcher and the way sex/gender as a variable is explored. Here, we examine, from a neurofeminist perspective, both questions: whether sex/gender identity is related to the examination of sex/gender as a variable and whether different approaches towards examining sex/gender are being used in different topics of study within neuroscience. Using the database of submitted posters to the Organization of Human Brain Mapping 2022 annual conference, we identified abstracts examining a sex/gender-related research question. Among these *target abstracts*, we identified four *analytical categories*, varying in their degree of content-related complexity: (1) *sex/gender as a covariate*, (2) *sex/gender as a binary variable for the study of sex/gender differences*, (3) *sex/gender with additional biological information*, and (4) *sex/gender with additional social information*. Statistical comparisons between sex/gender of researcher and the target abstract showed that the proportion of abstracts from *Non-binary* or *Other* first authors compared to both *Women* and *Men* was lower for all submitted abstracts than for the target abstracts; that more researchers with sex/gender-identity other than man implemented analytical category of *sex/gender with additional social information*; and, for instance, that research involving cognitive, affective, and behavioural neuroscience more frequently fit into the *sex/gender with additional social information*-category. Word cloud analysis confirmed the validity of the four exploratorily identified *analytical categories*. We conclude by discussing how raising awareness about contemporary neurofeminist approaches, including perspectives from the global south, is critical to neuroscientific and societal progress.

**Supplementary Information:**

The online version contains supplementary material available at 10.1007/s00429-023-02750-8.

## Introduction

### Sex/gender in neuroscience

For a long time, “sex omission” was standard practice in biomedicine, whereby sex/gender-related data were collected and analyzed as experimental variables, but the analyses were never reported in publication (Mamlouk et al. 2020). This may be one of the reasons why, in 2014, the NIH called for the inclusion of “sex as a biological variable” at all levels of analysis in biomedical research. However, this was interpreted by most research teams as a necessary comparison between binary sex/gender groups, i.e. the search for and publication of sex/gender differences (Richardson et al. 2015; Joel et al. 2015). The study of sex/gender in the brain is unique due to its great interest within and outside science (Rippon 2019), especially as neuroscientific outcomes have been shown to shape social opinion and ultimately the understanding of individuals as “cerebralized” persons (Ortega and Vidal 2007). When sex/gender is operationalized in neuroscience research, examinations have shown how notions, i.e. the ways we think of women or men [1] and of sex/gender, interfere with scientific results (Fine 2017; Bryant et al. 2019). For example, Fine (2013), Maney (2016) and Rippon et al. (2014) have demonstrated that research design and analysis are biased by sex/gender-related stereotyping, even prior to interpreting study findings. The question remains as to whether the NIH, with its initiative to foster sex/gender research, has exacerbated the focus on sex/gender differences and, thus, the reinforcement of binary (stereotyped) sex/gender notions in the neuroscientific research setting. Thus, whether this binary reinforcement led to increased neurosexism [2] or whether it permitted more nuanced approaches to sex/gender-related research remains an open question (Shansky & Woolley 2016; Garcia-Sifuentes and Maney 2021).

### Sex/gender diversity and the examination of sex/gender in neuroscience

While the relation between the sex/gender of the researcher and scientific research practices, such as publication and participation rates in the academic field, have been thoroughly examined (Son and Bell 2022; Ní Laoire et al. 2021), less interest has been focused on the relation between sex/gender of the researcher and whether or how sex/gender as a variable is considered in research. One of the few studies, a large data-based analysis in the field of biomedicine, found that women’s authorship is related to the likelihood of a study including sex and gender in the analysis (Nielsen et al. 2017). Nielsen and colleagues further demonstrated that women's participation in research is closely linked  to gendered health outcomes, showing the impact that diverse participation in medical research has on sex/gender equity. Relatedly, the relation between sex/gender of the researcher and the sex/gender variable seems to be of further interest when taking into consideration that women and sex/gender diverse researchers often use alternative approaches to examine sex/gender. For neuroscience, this was shown by Llaveria Caselles (2021), who additionally showed that these alternative approaches were delegitimized by male colleagues, leading to a discreditation of women or sex/gender diverse researchers in neuroscience. This discreditation was called a “testimonial injustice against the epistemic agents of alternative approaches” (p. 14). However, does the use of alternative approaches primarily by women and sex/gender diverse researchers justify making the research question a “women's research issue” [3] in neuroscience and beyond (Kaiser 2018), or should it be a shared research responsibility? And are these “alternative approaches” used by women and sex/gender diverse persons actually addressing the topic of sex/gender in the brain in a more complex, less conservative way and more aligned with contemporary knowledge on the interdisciplinary complexity of the sex/gender question?

### More diversity equals more objectivity

Regarding the sex/gender of the researchers within a field, the overrepresentation of one sex/gender identity may lead to a homogeneous and thus biased perspective. Science studies and feminist epistemology have shown that—exactly because objectivity is a “social achievement”—the more socially “complete” the science community is, the more complete, and thus, more objective the examination of the research questions must be (Longino 1990). Here, by drawing on these feminist accounts of scientific objectivity, we expect the different sex/gender identities to make a difference. A socially diverse representation of researchers in the examination of a question will provide a more valid examination. Additionally, when the object of this examination is a sex/gender-related variable, i.e. a potential sex/gender difference or similarity in the brain or if the research question is related to a biological or social sex/gender-related mechanism, it is even more intriguing to examine whether there is a relation between heterogeneous sex/gender researchers and the way sex/gender is investigated. This evidence on the social embeddedness of objectivity is linked to issues of inequality in neuroscience. The structural systematic disadvantageous treatment of women in neuroscience (Schrouff et al. 2019), for instance, may be weaker if diversity was larger.

### OHBM

In the present study, we turn to the international Organization for Human Brain Mapping (OHBM) to explore sex/gender diversity in neuroscience. OHBM is a multidisciplinary organization that aims to advance the understanding of the structure and function of the human brain through neuroanatomical, physiological, and psychological investigations. In 2022, more than half of the featured keynotes at the annual OHBM conference were women from different cultural backgrounds. This is a very particular case of sex/gender equality in STEM. This evolution in featured keynote speakers was made possible by the OHBM *Diversity and Inclusivity Committee*. Since 2019, OHBM's *Diversity and Inclusivity Committee* has put effort into prioritizing and emphasizing inclusion, sex/gender equality, and diversity (Tzovara 2021). Since the formation of the *Diversity and Gender Task Force* in 2016, the OHBM has dedicated considerable attention to the issue of equality in its community. Many activities have been implemented and a large number of specific goals that promote diversity and inclusivity have been achieved, such as the creation of a code of conduct, the introduction of the *Diversity Symposium* to the annual conferences, the application of selection criteria, “diversity of presenters” for keynotes, and the use of pronoun stickers on name badges. Due to the active evolution in this particular biotopic of neuroscience, it may be of interest to examine if the research being conducted at OHBM parallels the diversity efforts of the organization. Although a cause-and-effect relationship or a statistical association cannot be demonstrated in this type of analysis, our exploratory examination may reveal similar and interesting tendencies between diversity efforts and neuroscientific research.

The strong interdisciplinary character of OHBM as a neuroscientific conference demonstrates that the same research question can be addressed very differently (Boon 2020). This may result in distinct examinations of the sex/gender variable depending on the topical orientation of each subdiscipline (Schellenberg 2019). For example, molecular neuroimaging (Martins et al. 2021) may refer to the variable of sex/gender based on genetics, whereas in social affective neuroscience, the notion of a “social gender” may influence the implementation of measures that take the social concept into account. In the context of this study, we, therefore, ask: are different research areas in our neuroscientific site of knowledge production using different approaches to sex/gender?

Taken together, we draw on the ways of examining sex/gender, a more complete perspective to research questions, and OHBM’s efforts to foster equality to examine whether or not sex/gender diversity of scientists is related to conceptualisations of sex/gender in science. To address this issue, we analyze research material presented at OHBM annual conference in Glasgow in June 2022. In particular, this work examines three different questions:

- First, it compares the distribution between first authors' sex/gender in abstracts in target (i.e. abstracts explicitly addressing the topic of sex/gender) versus all abstracts.
- Second, it explores the target abstracts regarding their “level of complexity” in the examination of sex/gender, and how this is associated with first authors’ sex/gender.
- Third, it looks at the different approaches to sex/gender in relation to the topics of research.

Although we apply quantitative statistics, the present study is of exploratory character; its aim is to unearth qualitative tendencies. Finally, the authors of this paper put this initiative in the broader context of issues and discussions that have been raised previously by neurofeminism (Bluhm et al. 2012; Schmitz and Höppner 2014). *Neurofeminism* bridges a critical feminist perspective with brain sciences (Bluhm et al. 2012; Schmitz & Höppner 2014) and does so by means of two different approaches. It either empirically creates neuroscientific evidence about sex/gender based on an informed theoretical background of sex/gender theory (e.g. van Anders and Dunn 2009, Kaiser 2009, Joel 2015, Bryant 2019, Sanchis-Segura 2022), or it does so by evaluating neuroscientific practices of knowledge production about sex/gender (Kaiser 2012; Fine 2013; Bluhm 2013; Jordan-Young 2010; Walsh and Einstein 2020; Llaveria Caselles 2021, Ciccia 2022, Rippon 2021) and its intersected dimensions (Kuria 2014; Roy 2018, Duchesne et Kaiser 2021), including research on inequality in neuroscience (Asplund and Welle 2018, Schrouff 2019). Against this backdrop, here, the starting point of analysis is the second approach.

## Methods

### Search strategy

**Fig. 1:**
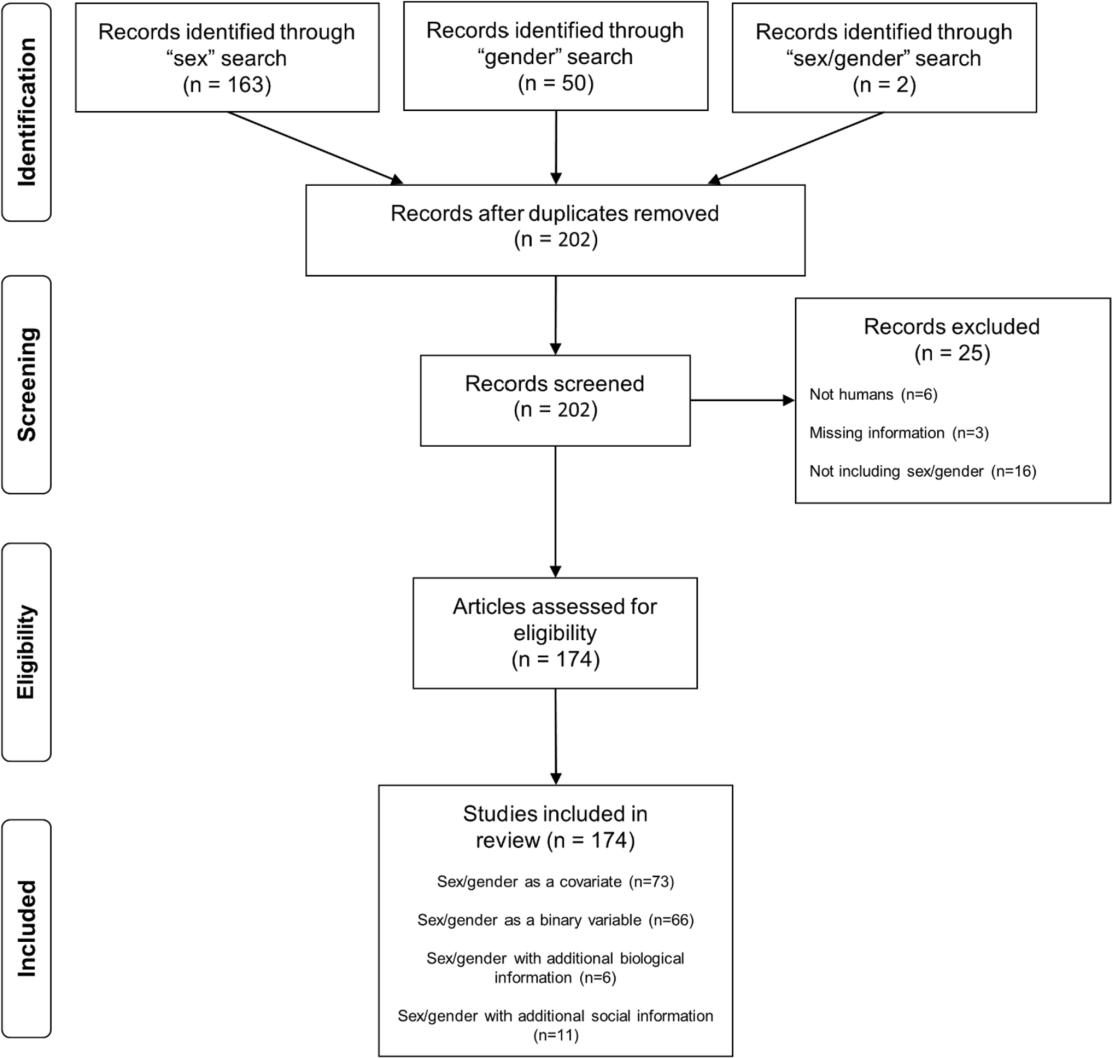
PRISMA flow diagram of the inclusion of abstracts in the review. Note: PRISMA = Preferred reporting items for systematic reviews and meta-analyses (http://www.prismastatement.org/)

To examine the research presented at OHBM 2022, a comprehensive electronic search was performed using the OHBM 2022 database for all poster abstract submissions in September 2022 (https://event.fourwaves.com/ohbm-2022/pages). The key search terms (in the title and/or abstract) were “sex”, “gender”, or “sex/gender”. Out of the 2140 submitted abstracts, the search identified a total of 215 abstracts approaching the topic of sex/gender (163 from the “sex” search, 50 from the “gender search”, and two from the “sex/gender” search); 202 abstracts after removing duplicates. The title and abstract of these 202 articles were screened, after which 25 abstracts were excluded for not including human samples (*n* = 6), missing information (*n* = 3) or not addressing the topic of sex/gender (*n* = 16). This resulted in 174 pre-selected abstracts, and their corresponding posters when available, which were further assessed (Fig. 1).

### Abstract categorization

While assessing the 174 abstracts, we identified four different types, i.e. *analytical categories*, of implementation of sex/gender that varied in their degree of content-related complexity. The categories identified, starting with the lowest complexity, were: (1) *Sex/gender as a covariate*, which included abstracts using sex/gender as a covariate, abstracts mentioning that a sex/gender-matched sample was used, or abstracts including sex/gender as a variable to perform tests in predictive and machine learning models (for example: “A connectome biomarker for blood pressure: a predictive analysis in 31,367 UK Biobank participants”); (2) *Sex/gender as a binary variable* for the study of sex/gender differences, which was used in interaction with different topics of study (psychiatric or neurological diagnosis, ageing, etc.) (for example: “Diagnosis and Sex in Autism Spectrum Disorder Explored using Phenomics, Genetics, and Neuroimaging”); (3) *Sex/gender with additional biological information*, with abstracts including some biological dimensional measures related to sex/gender (e.g. hormones) (for example: “Sex Hormones & Medial Temporal Lobe: 7 T MRI Shows Volume Changes at Subregion Level over Menstrual Cycle”); and (4) *Sex/gender with a social conception of gender and/or additional social information* (referred to as “Sex/gender with additional social information” from here on), with abstracts where the social component of sex/gender was the main topic of study (e.g. sex/gender stereotype threat), or where this was explored in interaction with other topics (e.g. language processing) (for example: “The Impact of Minority Stress on the Developing Brains of Gender Diverse Youth”) [4].

Moreover, in addition to the *analytical categories*, we additionally described the content of the 174 abstracts with regards to the topic of study. To this end, we considered the topic labels used by the OHBM. Since the topic labels from OHBM were too numerous, sometimes too specific (e.g. “PET”, “subcortical structures”), and in some cases not entirely distinctive, we resynthesized and restructured them resulting in our own 9 *topical categories*: (1) *Modeling and analysis methods*; (2) *Neurological and neurodegenerative disorders* (including Parkinson, Alzheimer, traumatic brain injury, etc.); (3) *Neurodevelopmental and disruptive behaviour disorders* (attention-deficit/hyperactivity disorder, autism, etc., as well as abstracts examining a factor that possibly had a detrimental influence in neurodevelopment (e.g. famine and immune system studies)); (4) *Lifespan developmental* (including healthy ageing); (5) *Psychiatric disorders* (depression, anxiety, schizophrenia, etc.); (6) *Cognitive, affective and behavioural neuroscience* (CABN); (7) *Brain anatomy* (when not including neurological, neurodevelopmental or psychiatric samples); (8) *Neurophysiology* (perception, sleep and homeostatic processes); and (9) *Other*.

Abstract categorization along the *analytical* and *topical* dimensions were performed by three independent reviewers (authors) resulting in an intra-class correlation coefficient (ICC) of 0.947 for the *analytical* and 0.713 for the *topical categorization*. In case of disagreement, the category chosen by at least two reviewers was kept, and in those cases where all three reviewers selected different categories, disagreements were resolved through clarification among raters. See Supplementary *Table 1* for the final categories assigned to each abstract, and other relevant information.

### Sex/gender classification of first author

One of the outcomes of the creation of the *Diversity and Gender Task Force* 2016 was the decision to collect information on sex/gender of participants in the registration and submission process at OHBM's annual conferences. Here, we built on this initiative and, relying on the member engagement office at OHBM, we obtained statistics for 2124 abstract entries [5] (after removing 9 duplicates) regarding the sex/gender and research affiliation country of the first author. As standardized in the last years, participants were invited to declare their sex/gender identification during the online registration process with the prompt “I identify my gender as …”. This sentence was followed by five options, one below the other, in the order “male, female, non-binary, other, prefer not to answer.” If participants selected “other,” they had the option to specify more information. Additionally, participants were asked to indicate the pronoun that should be used to address them in a blank field, but this information was not used in the analyses of the present study.

For subsequent analyses, the “prefer not to answer” replies were considered missing (18 participants), while the “non-binary” and “other” replies were put together in a *Non-binary/Genderqueer/Other* classification which included people who self-described as genderqueer [6]. We are aware that through this classification valuable information on the fluency and ambiguity of sex/gender and complexity of all identities gets lost, yet this approach was resorted to for pragmatic reasons. To us, this classification is important as a first approximation to know the representation of people who did not identify their sex/gender as “male” or “female”, and it was not intended to make the categories exhaustive. In this sense, we refer to a *Woman* or to a *Man* if the person identifies as "female" and "male", which were the terms used by the OHBM online registration question, see above*.* We assume that the trilogy *Non-binary/Genderqueer/Other* does not contain trans women and trans men, who we expect to have marked the category “female” or “male”, respectively (as it involves both cis and trans populations). Thus, we used the following three categories for the statistical analyses: (1) *Non-binary/Genderqueer/Other* (NB/GQ/O); (2) *Woman*; and (3) *Man*.

### Statistical analyses

Statistical analyses were performed using IBM SPSS Statistics 21 (IBM, USA). In a first step, we explored the distribution of the first authors’ *sex/gender classification* (i.e. *Non-binary/Genderqueer/Other*, *Woman*, *Man*) in the 156 *target abstracts* (174–18 missing and/or “prefer not to answer” sex/gender classifications) as compared with the 1953 *all abstracts* ((2124–171 missing and/or “prefer not to disclose”) (see Table 1). To examine who is studying sex/gender and how it is studied, we then focused on the target abstracts, exploring first whether the four *analytical categories* identified in the section Abstract categorization (i.e. *sex/gender as a covariate*, *sex/gender as a binary variable*, *sex/gender with additional biological information*, *sex/gender with additional social information*) were differently distributed depending on a) the first author’s sex/gender classification and on b) the *topical categorization* (i.e. *Modeling and analysis methods*; *Neurological and neurodegenerative disorders*; *Neurodevelopmental and disruptive behaviour disorders*; *Lifespan developmental*; *Psychiatric disorders*; *CABN*; *Brain anatomy*; *Neurophysiology*; *Other*). To analyze each possible paired comparison between *abstract type* (target or all)  and *first authors’ sex/gender classification*, between *analytical category* and *first authors’ sex/gender classification*, and between *analytical category* and *topical categorization*, respectively, post hoc testing was applied using the corresponding Bonferroni adjustment. Geopolitical [7] information was obtained by collecting the countries in which the university of affiliation provided by the first author were located.

**Table 1:**
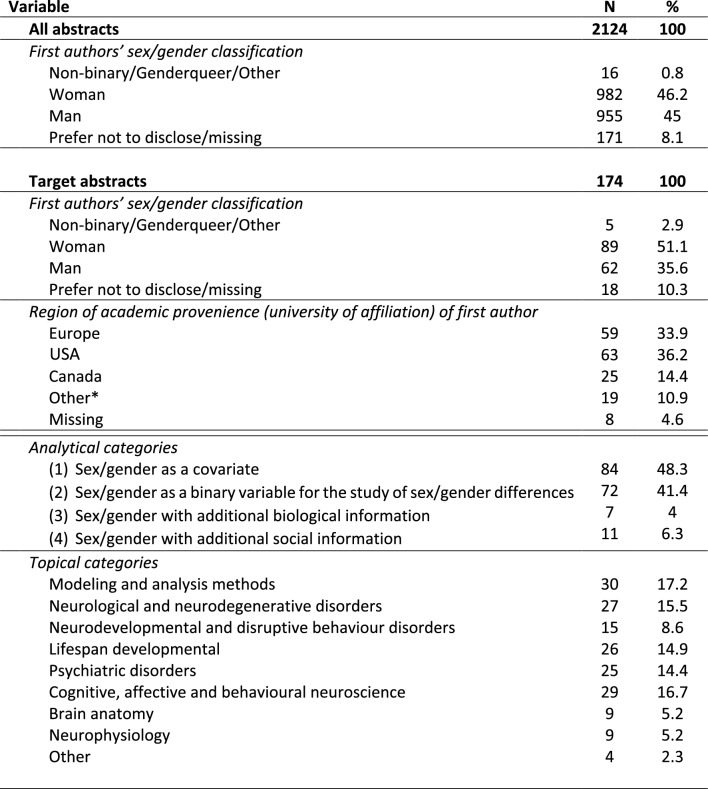
Abstracts characteristics according to different information explored in the study

*Note:* Base of this table are the number of all abstracts *N* = 2124 and the *N* = 174 of pre-selected target abstracts (and not the 1953 and the 156 that entered statistics after subtracting 171 and 18 missing items, respectively). *Other countries were: 3 Republic of Korea, 3 Taiwan, 2 Australia, 2 Mexico, 2 Hong Kong, 2 China, 1 Japan, 1 New Zealand, 1 Israel, 1 Islamic Republic of Iran, and 1 Brazil

### Word cloud generation

Further, to validate the classification by sheding semantic light onto the question of how sex/gender is approached within all four *analytical categories*, the associative conceptual character of each of these categories was assessed by word cloud analysis. For this aim, a free word cloud generator was applied (https://www.freewordcloudgenerator.com/generatewordcloud). We first focused on the title of the abstracts to explore the concepts addressed in each category. Word clouds representing the ten most frequent words for the titles of each of the four *analytical categories* (i.e. *sex/gender as a covariate*, *sex/gender as a binary variable*, *sex/gender with additional biological information*, *sex/gender with additional social information*) were thus created. In a second step, we explored the semantics and associated terms for the three *analytical categories* that addressed sex/gender as a scientific question (*analytical categories 2*, *3*, and *4*) and excluded *analytical category 1* in which sex/gender was not scientifically but “technically” addressed. For this latter approach we used the whole abstract as an input and generated 50-word clouds for each of the three categories. To avoid irrelevant words for the word clouds, the following terms were deleted from the input text files before creating the word clouds: “analysis”, “conclusion”, “data”, “fig”, “figure”, “found”, “measures”, “methods”, “observed”, “result”, “results”, “show”, “shown, “studies”, “study”, “using”.

## Results

### Descriptives

General qualitative distributions (Table 1) show that 0.8% of all first authors describe themselves as *Non-binary/Queer/Other*, 46.2% as *Women*, and 45% as *Men*, while 8.1% individuals chose “prefer not to disclose” the sex/gender question. Within *target abstracts* (*N* = 174), only 10.9% of the first authors came from countries that were not Europe, USA and Canada. Regarding the analytical approach, the most common was sex/gender as a covariate (48.3%) followed by sex/gender as a binary variable (41.4%). Regarding topical categories, the highest percentages (14.4–17.2%) were *Modeling and analysis methods*, *Neurological and neurodegenerative disorders*, *Lifespan developmental*, *Psychiatric disorders,* and *CABN*.

### Statistical analyses

#### First authors’ sex/gender classification in all abstracts vs. target abstracts

Pearson chi-squared test showed a significant result (*χ*^*2*^ (2, *N* = 2109) = 12.073, *p* = 0.002) demonstrating that there is a relation between factors sex/gender of first author and abstract group membership (target- versus all-group). The effect size shows that this relation is weak (*Cramer's V* = 0.08, *p* < 0.001). According to post hoc comparisons (Bonf.-corrected *p* < 0.05), for the factor group membership, the percentage of target abstracts from NB/GQ/O-first authors (3.2%) differed from the percentage in all abstracts from NB/GQ first authors (0.8%), and the percentage of target abstracts from men (39.7%) differed from the percentage of all abstract in men (48.9%). For the factor of sex/gender, a significantly higher percentage of target abstracts had NB/GQ/O-first authors (23.8%) compared to women (8.3%) and men (6.1%) first authors, resulting in a significantly lower percentage of all abstracts with NB/GQ/O-first authors (76.2%) compared to women (91.7%) and men (93.9%) first authors (Table 2).

**Table 2:**
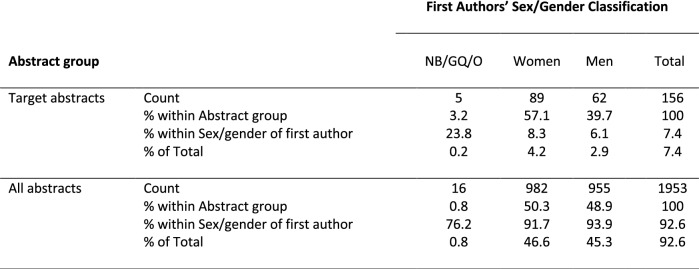
Abstracts distribution according to first authors’ sex/gender classification and abstract group

*Note:* For the underlying post-hoc testing the missing- and “prefer not to disclose”-items were taken off, resulting in *N*_All_ = 1953 (2124_All with Missing/Prefer-not-to-disclose_–171_Missing/Prefer-not-to-disclose_) and in *N*_Target_ = 156 (174_Target with Missing/Prefer-not-to-disclose_–18_Missing/Prefer-not-to-disclose_). “Count” refers to the actual number of individuals, “% within” describes the percentage of the “Count” of the correspondent row

#### Analytical category versus first authors’ sex/gender classification

**Fig. 2:**
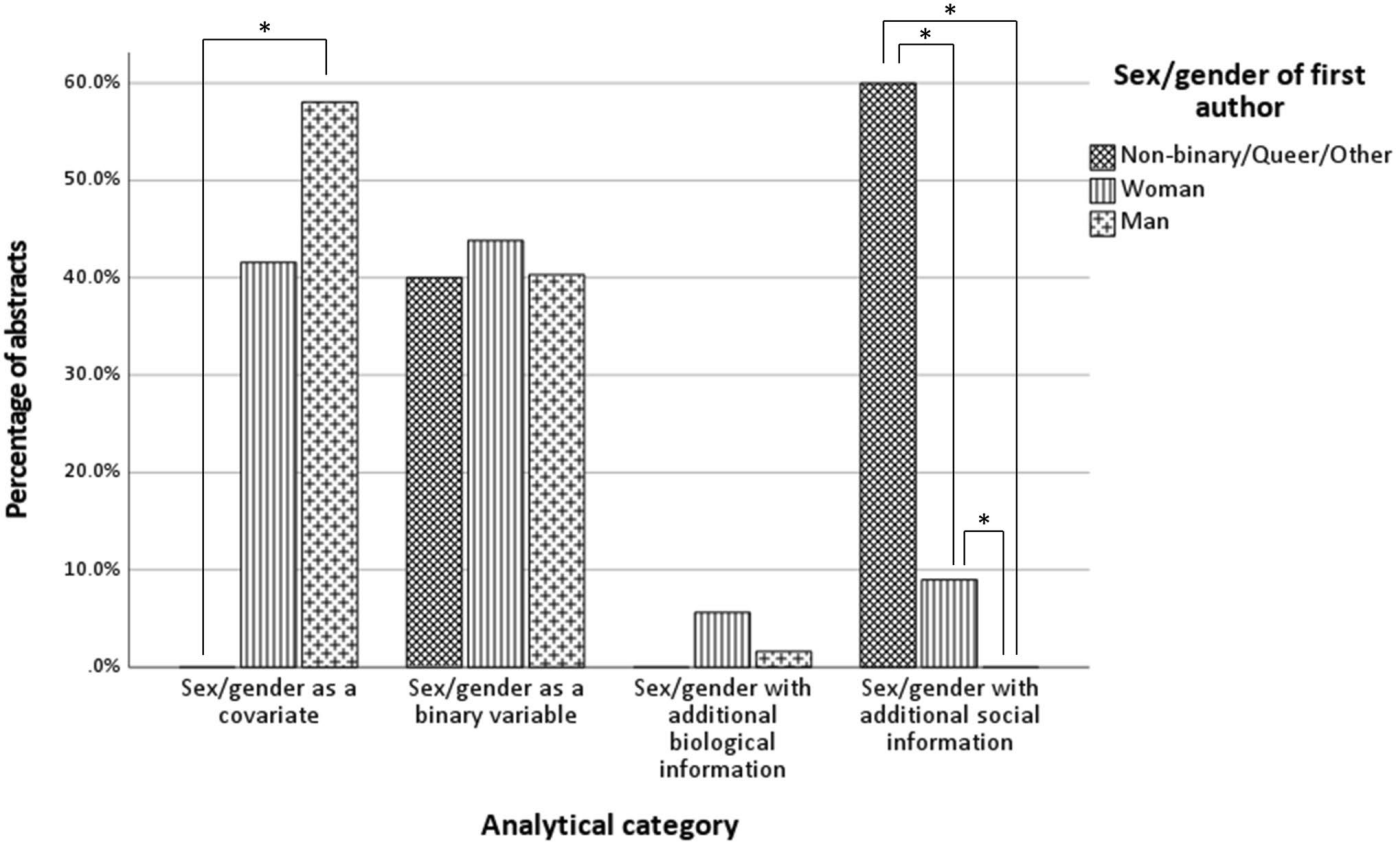
Overview of first abstracts’ distributions. Distribution of abstracts for each *analytical category* by *first authors’ sex/gender classification*. *Significant at *p* < 0.05 (Bonf.)

For target abstracts, the chi-squared test between the factors analytical categories and the sex/gender of first authors was significant, *χ*^*2*^ (6, *N* = 156) = 31.101, *p* < 0.001. The effect size was 0.32 (*Cramer's V*, *p* < 0.001). Post hoc comparisons revealed that for the first authors’ sex/gender-factor there was a greater proportion of abstracts of the first analytical category (sex/gender as a covariate) in men (58.1%) compared to NB/GQ/O-first authors (0%). There were similar values for analytical categories 2 (sex/gender as a binary variable; ~ 40%) and 3 (sex/gender with additional biological information; < ~ 5%) for all three sex/genders of first author groups. Finally, the highest rates of abstracts from analytical category 4 (sex/gender with additional social information) were present among first authors self-described as NB/GQ/O (60%), followed by women (9%), and finally men (0%) having the lowest rates of abstracts in this analytical category (Fig. 2) (Table 3). When looking at the post hoc tests for factor analytical category, abstracts by women were equally distributed across all four analytical categories (covariate: 50.7%, categorical: 59.1%, biological: 83.3%, social 72.7%), while men had a higher proportion of abstracts in the first analytical category compared to analytical category 4 (covariate: 49.3%, social: 0%), and NB/GQ/O first authors presented a higher proportion of abstracts in analytical category 4 (27.3%) compared to both analytical categories 1 (0%) and 2 (3.0%) (Table 3).

**Table 3:**
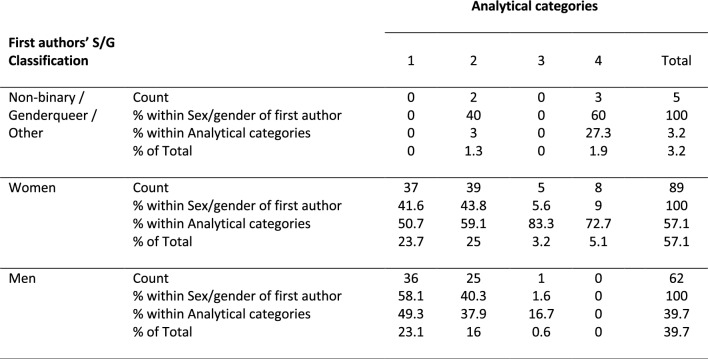
Abstracts distribution according to first authors’ sex/gender classification and analytical category in target abstracts

*Note:* Analytical categories: (1) Sex/gender as a covariate, (2) Sex/gender as a binary variable for the study of sex/gender differences, (3) Sex/gender with additional biological information, (4) Sex/gender with additional social information

#### Analytical category versus topical categorisation

**Fig. 3:**
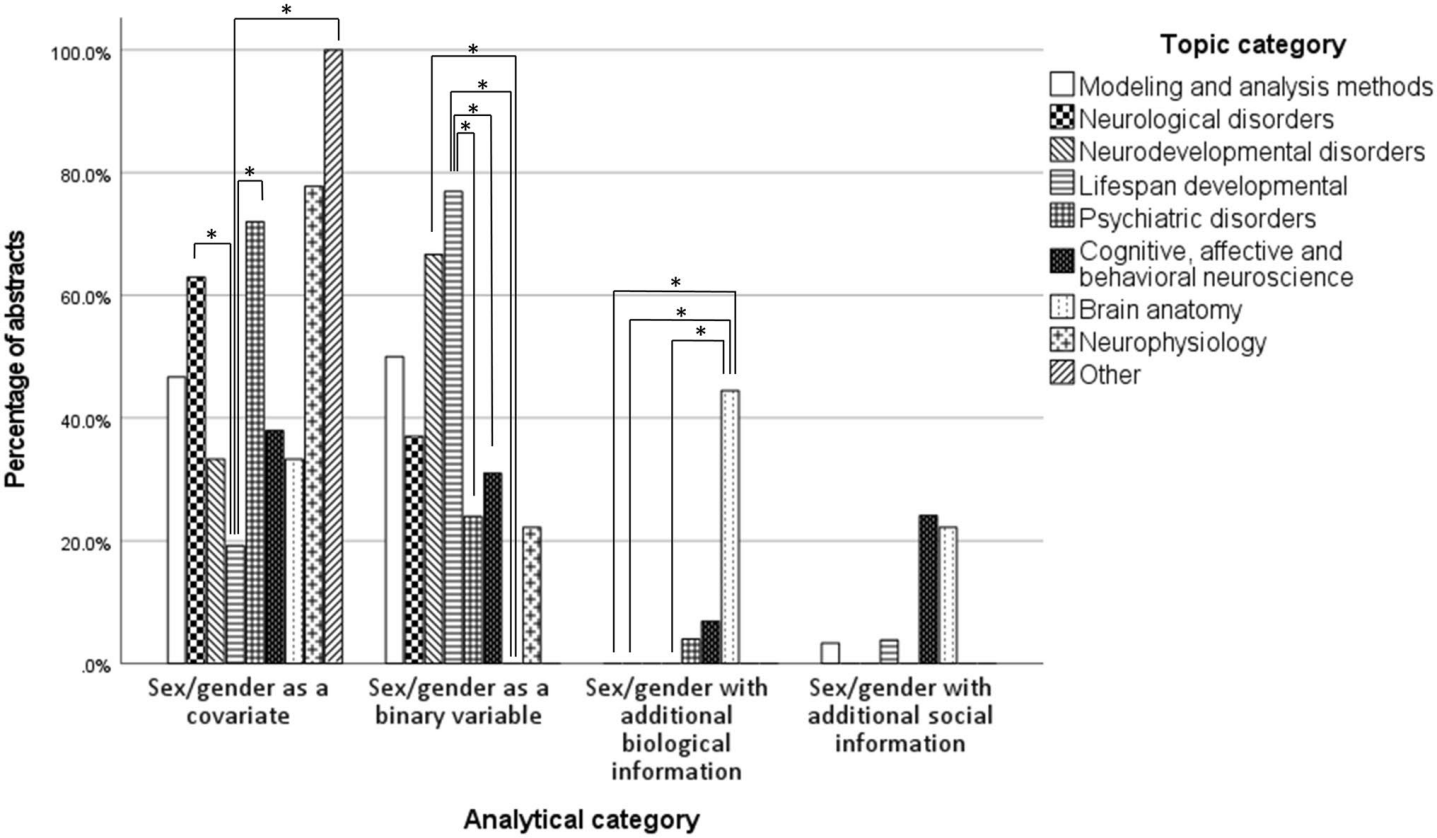
Overview of first abstracts’ distributions. Distribution of abstracts for each *analytical category* by *topical category*. *Significant at *p* < 0.05 (Bonf.)

Finally, the chi-squared test between analytical and topical categories was also significant, *χ*^2^ (24, *N* = 174) = 99.45, *p* < 0.001. The effect size was 0.44 (*Cramer's V*, *p* < 0.001). Post hoc comparisons for factor topical categories revealed a lower rate of *Lifespan developmental* abstracts using the first analytical approach (sex/gender as a covariate) (19.2%) compared to *Neurological disorders* (63%), *Psychiatric disorders* (72%), and *Other* (100%) abstracts, while there was a higher rate of *Lifespan developmental* abstracts using the second analytical approach (sex/gender as a binary variable) (76.9%) compared to *Psychiatric disorders* abstracts (24%), *CABN* (31%), and *Brain anatomy* abstracts (0%). Moreover, the proportion of *Neurodevelopmental* *disorders* abstracts (66.7%) within this binary analytical category was higher than that of  *Brain* *anatomy* abstracts (0%). Within the third analytical category (sex/gender with additional biological information), there was a higher proportion of *Brain anatomy* abstracts (44.4%) compared to the *Modeling and analysis methods* (0%), *Neurological disorders* (0%), and *Lifespan developmental* abstracts (0%), and finally, for analytical category 4 (sex/gender with additional social information) there was an equal proportion of abstracts for all topical categories (Table 4). Then, when looking at the post hoc tests in the factor analytical categories, abstracts from *Modeling and analysis methods*, *Neurological disorders*,  *Neurodevelopmental disorders*, *Psychiatric disorders*, *Neurophysiology* , and  *Other*, were equally distributed across all four analytical categories (ranging from 0 to 20%). On the other hand, *Lifespan developmental* abstracts more frequently used the second analytical approach (27.8%) compared to the first (6.0%); *CABN* abstracts more frequently used the fourth analytical approach (63.6%) compared to both the first (13.1%) and the second analytical approaches (12.5%); and finally, the rate of *Brain anatomy* abstracts was higher for the third analytical category (57.1%) compared to both the first (3.6%) and the second (0%), while it was also higher for the fourth analytical category (18.2%) compared to the second (Table 4*, *Fig. 3).

**Table 4:**
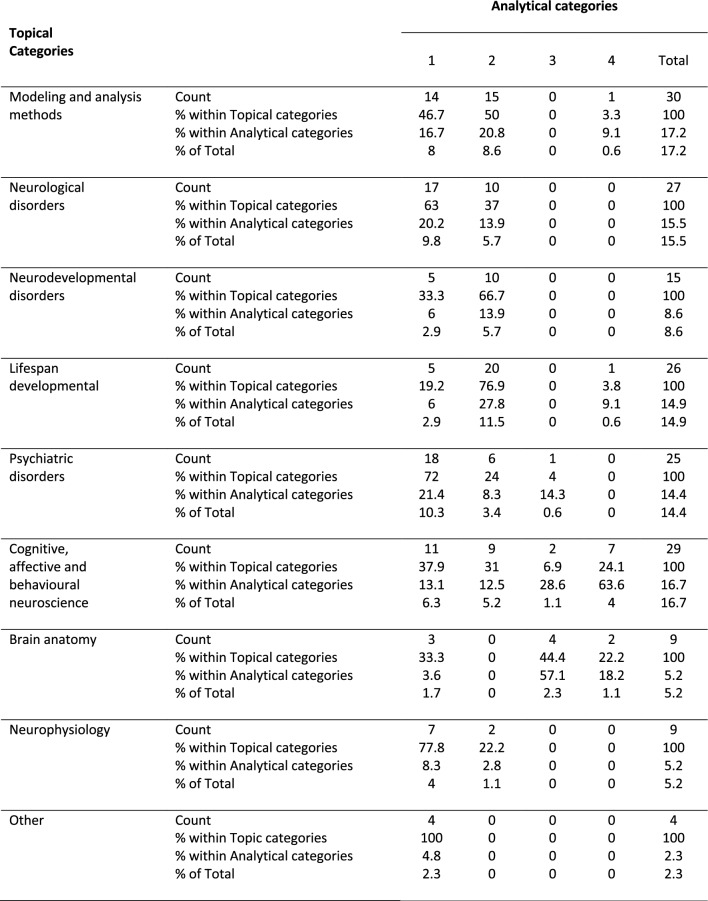
Abstracts distribution according to analytical category and topical category in target abstracts

*Note:* Analytical categories: (1) Sex/gender as a covariate, (2) Sex/gender as binary variable for the study of sex/gender differences, (3) Sex/gender with additional biological information, (4) Sex/gender with additional social information

### Semantics of analytical categories

**Fig. 4:**
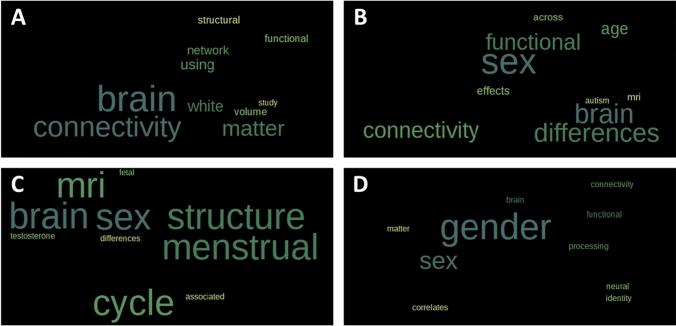
Semantics of the four different analytical categories examining sex/gender. Word clouds from abstract titles (10 most frequent words) in all four different *analytical categories*: **A** Sex/gender as a covariate, **B** Sex/gender as binary variable for the study of sex/gender differences, **C** Sex/gender with additional biological information, **D** Sex/gender with additional social information

**Fig. 5:**
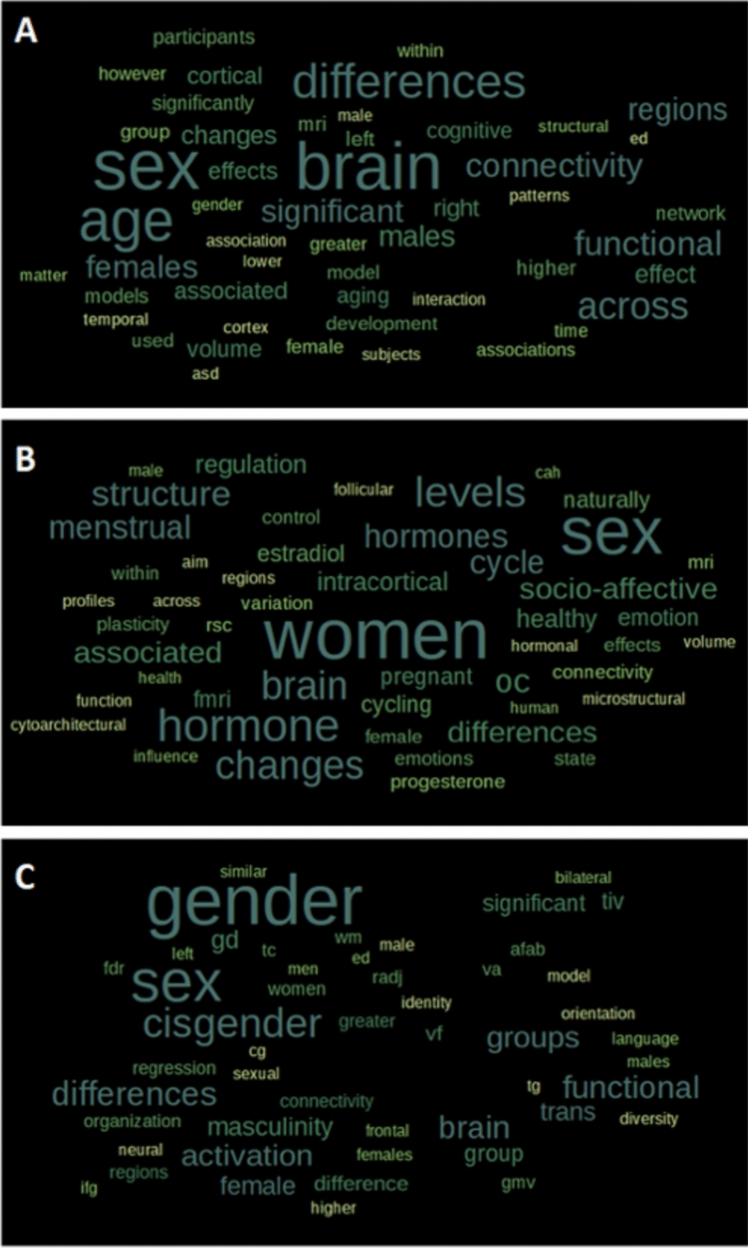
Semantics of three of the four different analytical categories examining sex/gender. Word clouds (50 most frequent words) generated from the content of the abstracts of the analytical categories. **A** Sex/gender as binary variable for the study of sex/gender differences, **B** Sex/gender with additional biological information, **C** Sex/gender with additional social information

Word clouds reveal that the most frequent terms from the titles have patterns that are clearly aligned with the definition of our four *analytical categories* (Figs. 4, 5), thus validating these categories. First, the concept of “gender” appears specifically in the fourth category while neither “gender” nor “sex” appear in the first category. Paralleling our observation of binary sex/gender classification (category 2) and biological use of sex/gender (category 3), the second and third word clouds focus on the term “sex”. Furthermore, “differences” appears as a frequent term in the second *analytical category*—described by us as an approach focusing on dissimilarity between women and men. Interestingly, “differences” appears as well, but to a lesser extent, in the third *analytical category* but not in the fourth category. Finally, terms referring to body biology (such as “menstrual” and “testosterone”) appear specifically in the third category, while the term “identity” appears specifically in the fourth category. Thus, overall, the titles of the abstracts correspond to the *analytical category* to which the latter were assigned. To zoom in further and investigate the semantics and concepts of interest that characterize the studies addressing sex/gender as a scientific matter, we examined the word clouds generated based on the abstracts for the *analytical categories 2*, *3*, and *4*. These word clouds hence suggested that the second category mainly use terms pertaining to a biological background (“female” and “male”), although not specifically measuring sex/gender as a biological variable and tend to emphasize differences with the frequent use of terms like “brain”, “sex”, “differences”, “greater”, “higher”, “pattern”, “effect”, “significant”. As expected, the semantics framework of the third category was importantly pertaining to biology with terms including “brain”, “women”, “sex”, and “hormone”, but also terms such as “socio-affective” and “emotions”, due to two specific abstracts exploring these topics. Finally, the semantics framework of the fourth category appears relatively diverse including terms such as “gender”, “sex”, “cisgender”, “groups”, “differences”, “masculinity”, “activation”, and “functional”.

## Conclusions

### Summary

For over 30 years now, measures for sex/gender equality in STEM have been implemented. Structural changes for more inclusion have been achieved, however, inequality, for instance in neuroscience, still exists (Schrouf et al. 2019). Seeing that institutions such as OHBM are dedicating so much resources into inequality and diversity efforts, we, as part of the OHBM community, aimed to present our findings related to the diversity of authors and the research they do. Relying on feminist epistemology, we state that the overrepresentation of one sex/gender identity leads to a homogenous and, thus, incomplete perspective to a research question. The role of those who produce knowledge and the context of the authors, including their identity, directly influences the way neuroscientific topics are understood, studied, and how the results are interpreted. Accordingly, we focused on the diversity of researchers and their relation to sex/gender as a variable, as well as the type of scientific research in which sex/gender is being examined. Our results furnish support to a downstream benefit for neuroscience and the OHBM community. This study cannot prove causation between OHBM´s diversity efforts and knowledge production, rather, the presented exploratory outcomes may be the frame for building new hypotheses and may foster further discussion about what it means to host a diversity-friendly conference. Additionally, our data may unearth and inspire new research innovation for exploring the specific combination of diversity in researchers and sex/gender-related knowledge production.

When looking at how abstracts focusing on sex/gender as a variable (target abstracts) versus the entirety of submitted abstracts (all abstracts) differ from each other, we found that diversity (*NB/GQ/O*-first authors) is larger in the former than in the latter and “maleness” (*Man* as first authors) is more common in all abstracts than in target abstracts. Focusing directly on the sex/gender of first authors as groups, it becomes clear that *NB/GQ/O-*first authors are, proportionally, more represented in the target abstracts than women and men, and conversely, less represented in all abstracts. Thus, although the size of the effect is small, these results reveal that the sex/gender of first authors is clearly related to interest in the sex/gender variable, suggesting that authors of abstracts that address sex/gender questions tend to be more diverse.

The medium effect found between *first authors’ sex/gender classification* and *analytical category* can also be interpreted as evidence for how diversity plays a role: there is a considerable link between the authors describing themselves as non-binary, genderqueer or as belonging to another sex/gender identity and the way they address the sex/gender question. When one looks at the rates of how sex/gender is examined between *NB/GQ/O*, *Women* and *Men*, the routine or “technical” way of using sex/gender, i.e. as a covariate, was mostly found in research first-authored by men—which differed clearly from the 0% found in the NB/GQ/O-group. Sex/gender as a binary and biological variable did not vary much in all other sex/gender groups, but in terms of understanding sex/gender as being partly social, NB/GQ/O-first authors were the most represented, followed by women, while men were not represented in this category. The representation of *NB/GQ/O*, *Women* and *Men* by the analytical categories also demonstrated a role for diversity in neuroscientific research. Women were equally distributed across all four analytical categories, while men had a higher proportion of abstracts in the “technical” compared to the “social” analytical category. Further, NB/GQ/O first authors presented a higher proportion of abstracts taking into account the social side of sex/gender compared to both analytical categories 1 and 3. Again, this shows how the identity of the researcher influences what is crucial for questions of sex/gender: the social context.

For the relation between analytical category and topical categorisation, there was a medium effect size demonstrating that fewer researchers in the non-medical or “social” fields of neuroscience, such as *Lifespan*, used the traditional covariate-option to address sex/gender, whereas more medical subdisciplines, like  *Neurology* or *Psychiatry*, used this analytical category to a greater extent. Conversely, there was a higher rate of *Lifespan developmental* abstracts using sex/gender as a binary variable compared to *CABN* showing that *CABN* seems to offer the opportunity for a less difference-based application of sex/gender as a variable. Interestingly, the social understanding of sex/gender was similar in all subdisciplines. When comparing the biological analytical approach to the covariate-approach, again *Lifespan developmental* abstracts were more frequently used. The most clear result supporting our hypothesis is that the “social” subdiscipline of *CABN* more frequently used the social analytical approach compared to both the covariate and the biological analytical approaches. The latter showed how different examinations of sex/gender as a variable resonate with the topical orientation of each subdiscipline (Schellenberg 2019).

Despite the representation of socially oriented subdisciplines at OHBM, the vast majority of work still relies on conservative scientific approaches to the topic of sex/gender. In other words, the most used analytical approach to sex/gender was by approaching it as a covariate (48.3%) followed by sex/gender as a binary variable (41.4%). This clearly reflects that sex/gender is analyzed to routinely “control” this variable instead of truly interrogating it, elsewhere described as examining sex/gender “as by-product” (Kaiser et al. 2009).

### Comments “from the margins”

Sex/gender inequality in neuroscience has also been demonstrated to exist in Latin America. Silva (2021), for instance, showed an overrepresentation of men at important professional grades, i.e. in full professorships, in the neuroscientific field. This overrepresentation of only one sex/gender at this crucial level of influence on research does not project the only incomplete perspective to a studied object. Also other aspects of researchers’ identity may influence scientific research, for instance, the geopolitical identity (Cuéllar Laureano 2015). In the study presented here, most participants were from Europe and the United States, with significant participation from Canada as well (Table 1). As already shown for the OHBM community (Tzovara 2021), these distributions demonstrate that further effort regarding inclusion is needed. Given the general experience from researchers from the Global South as “being researchers from the margins” (Spivak 1988), they are more used to understanding the notion of the “situatedness” of knowledge production (Haraway 1988); i.e. depending on your location and identity, you can be included or excluded in knowledge production. This makes clear how there are, globally speaking, specific implicit positions of influence at stake when we do science, neuroscience included.

There is a standard practice in the natural sciences fields of approaching our objects of research by questions such as “What is xy?”, “How does xy work?”. These approaches asking what things “are” and how they “work” are guiding principles in western science. In the case of sex/gender in neuroscience, this results in a brain “being” different between women and men—with little attention to issues of intersectionality (Crenshaw 1991, Blowleg 2008) or race and class (Subramaniam 2009). Producing knowledge from outside these guiding principles permits bringing new questions into the mainstream, questions such as “Why do we do that research on xy?” and “For whom do we produce xy?” (Heler 2010) that clearly differ from the guiding questions “What is xy?”, “How does xy work?”. If the knowledge we produce in neuroscience promotes the sex/gender biases or only uses guiding principles endorsed by scientific bodies of western neuroscience, it must be asked: whose interests is the field of neuroscience responding to? Standardizing the subjects of study along the sex/gender dimension (or not considering it at all), coupled with the fact that (1) sex/gender identity of the researchers is linked to the grade of complexity of the sex/gender approach and (2) newer subdisciplines address sex/gender in more complex ways, risks the reinforcement of close-meshed and Eurocentric perspectives in neuroscience research.

The questions remain open as to whether the topics of interest, as well as the type of complexity of the studies, is comparable between the Global North and the Global South. For example, between Europe and Latin America, since, although sex/gender inequalities are global, the forms of inequality are local. Decolonial perspectives would enrich these conversations (Lugones 2008, García Dauder 2022; Maffía 2008, Hammonds and Subramaniam 2003).

### Limitations of this study

Our classification criteria in (a) *Non-binary/Genderqueer/Other,* (b) *Woman,* (c) *Man* does not capture the complexity of identities, and perhaps such criteria implied concealing identities that were not cis women and cis men. For example, in the categories *Woman* and *Man* there could be trans women and trans men invisible in the present analysis. An additional cis–trans classification within the *Woman*–*Man* options would allow for exploring our research question further, and to better investigate the representation of non-normative identities in the production of knowledge. Based on our findings that NB/GQ/O researchers use more complex analysis, it is possible that further delineating the *Woman–Man* options into cis and trans classifications would provide greater nuance to the analyses. Unfortunately, the option of explicitly declaring a binary trans identity was not possible to consider here because we relied on previously acquired data that did not distinguish identities at that level.

In addition, another limitation is the “prefer not to disclose”-entries. It is hard to know the causes for participant’s choice of that option, but studies on effects of discrimination suggest that some people use that option to protect themselves from the possible effect of negative and hostile or hidden sexism and discrimination (Gurieva et al. 2022; Bosak and Sczesny 2011). This may result in more women or diverse people having reasons to do so.

Moreover, we would like to note that our abstract categorization was not exclusively based on methodological criteria. For example, although we named category 2 as “Sex/gender as a binary variable”, not all abstracts performing between-group comparisons were included in this category. Instead, what we meant with “Sex/gender as a binary variable” is that abstracts included in that category conceptualize sex/gender in a biologically binary way. Contrarily, abstracts were included in category 4 when they were based on a social conception of gender (regardless of the statistical analyses being performed). We realised this is one of many possible categorisations on how to approach sex/gender analyses, and acknowledge that different categorisations could have yielded partially different results.

Another limitation is that in this study the comparison of *sex/gender analytical categories* among researchers with *sex/gender identities* (resulting in, e.g. first analytical category in *Men* = 58.1%) may be confounded by other factors. For example, one possible confound could be that sex/gender identities may be differently distributed in the different neuroscience research topics, such as for instance more women or non-binary/other in CABN. This makes the different topics (or the interaction of topics with sex/gender identity) the real factor for the use of different sex/gender analytical categories. Due to the exploratory nature of this study and to the low numbers in the existing categories, we decided not to lose statistical power by performing this triple interaction, but future studies should look into this issue.

### Informed science

The benefits of diversity in science can be broad. The benefits can be ethical, such as having a fairer distribution of the social goods produced by scientific activity or questioning deadlocked assumptions and notions embedded into scientific questions. The benefits of diversity can also be epistemic, such as obtaining a greater objectivity through broader inclusivity (Longino 1990). Lastly, they can be social, such as deciding within the scientific community what questions should be asked, what is accepted as evidence, and what is considered a risk (Melo-Martín 2015). In our study, examples of the benefits of diversity in science include the more adequate approaches to the variable of sex/gender, i.e. approaches that go beyond the routine examination to sex/gender as “covariate” or “binary”. Moving away from the first and second analytical categories produces knowledge that serves values relating to sex/gender equality. In other words, when we overcome that sex/gender is nothing more than an invisible statistical variable, or when we overcome that sex/gender is taken as a fixed default-factor, then we can develop a multi-faceted, well-informed, changeable operationalization of sex/gender as a variable. These new operationalizations would support the values of sex/gender equality insofar as they allow for diversification and amplification of an understanding of intersectional sex/gender in research—and are both ethical and epistemic benefits to diversity.

It is crucial to keep in mind that emphasis on numbers alone will not solve the problem of the lack of diversity in (neuro)science. Also, changes to the way (neuro)science is applied and performed are needed, such as innovation in science education and/or changes in professional evaluation (Hussénius 2014). Additionally, alternative ways of understanding sex/gender in (neuro)science are required. Breaking up with stereotypes related to sex/gender, such as the “male” capacity for abstraction and reasoning, are considered necessary to do good science (Maffía 2008).

We conclude by advocating for the importance of neurofeminism in the production of neuroscientific knowledge. Diverse and interdisciplinary feminist approaches are critical to diminish sex/gender bias in terms of equality in the workforce as well as in neuroscientific research itself. Future research is needed to work towards knowledge based on interdisciplinary cooperations that can enable approaches that go beyond conventional WEIRD (Western, educated, industrialized, rich, and democratic) perspectives (Henrich 2010). In this line, the NIH as well as other funding agency initiatives could help broaden the way sex/gender research is being performed by expanding their recommendations. Instead of just asking for the inclusion of women and testing for sex/gender differences, they could stimulate a more complex approach to sex/gender that would ultimately reach society's understanding of the topic.

By declaring our own position as a neurofeminist perspective, we aim to produce this type of less sex/gender-biased knowledge.

#### Take home message

Diversity plays a role in scientifically addressing sex/gender or not, the way sex/gender is scientifically addressed, and the topic chosen. The main findings from our statistical comparisons are threefold: (1) non-binary and sex/gender identities other than women and men are more often found in research on sex/gender; (2) more researchers with sex/gender identity other than *Man* implement the more complex analytical category for sex/gender; and (3) researchers involved in cognitive affective & behavioural neuroscience more frequently used a complex way of addressing sex/gender.

#### Footnotes

1. Since we "defend" that sex/gender identity is not a biological fact, defined genitally, but an experience that transcends anatomical/physiological data, we refer to the categories of “women” and “men” not as natural classes or categorizations. We further consider those to belong to these categories who identify themselves by these terms. Moreover, note that when we cite literature we cannot reconstruct in all cases whether it refers to women in a self-defined sense or whether they may distinguish between trans women and cis women, or trans men and cis men, respectively. Thus, unless it was a study that explicitly focused on the topic of trans or cisgender or it was a study that explicitly let the reader reconstruct this information, we here use the terms woman or man to denote the uncertainty that prevails in science.
2. Neurosexism is defined as the creation or reinforcement of sex/gender stereotypes and sexism through neuroscientific practices and their results (Fine 2013).
3. When talking about women, men or diverse people in this paragraph, we are not referring to ahistorical and pan-cultural traits. We consider these differences to be a constitutive part of sex/gender norms, and not the result of any causal relationship to reproductive possibilities.
4. The main difference between category (4) and category (2) is that in (2) only group comparisons with sex/gender as a binary variable were considered (in interaction with whatever topic), while in (4) sex/gender was taken into account as a “social” variable, regardless of its possible interaction with any other topic. Nonetheless, abstracts performing group comparisons could also be included in category 4, as long as they used a social conception of sex/gender (e.g. classifying participants based on sex/gender identity instead of—or in addition to—biological sex/gender).
5. Consider that the number of abstracts *N* = 2133 obtained from the engagement office is not consistent with *N* = 2140, the number of abstracts obtained from the platform of the conference. For statistical analyses, *N*_Total_ = 2124 was applied, see section “Sex/gender classification of first author”.
6. The *Non-binary/Genderqueer/Other* group consists of 15 *Non-binary*, 2 women-*Queer*, 2 *Other*-prefer to self-describe as "male", and 1 *Other* (with no further specification) entries. Regarding the *Other*-category, it seems crucial to mention that those who marked *Other* are doing so from a place of enunciation that is always related to the sex/gender binary system which constitutes a fundamental element of our identity (although normative and not natural). The reason why we cannot identify ourselves as "other" like anything outside this system is because there are no categories that are in the framework of intelligibility that makes us persons outside this logic (Butler 1990).
7. “Geopolitics” has been introduced in the frame of emancipatory analysis of the Global South. The term “geopolitical” describes how it is never only the geographical location of a country and its name (f.i. “Uruguay”) but also a global political position (by terms of global power relations) that is expressed when we apply country names  describing global geographical locations. Thus, the Global South is for instance just more than countries lying in the geographical location of the southern hemisphere, the Global South implies a history of colonialism, of extractivism, of dominance by “northern” countries. These lines of thoughts are important to our discussion in the conclusion of this article.

### Supplementary Information

Below is the link to the electronic supplementary material.

Supplementary file1 (DOCX 60 KB)

## Data Availability

The datasets generated during and/or analysed during the current study are available in the supplementary data, added below.
