# Supplementary material for: Researchers' sex/gender identity influences how sex/gender question is investigated in neuroscience: an example from an OHBM meeting
Source: Brain Struct Funct. 2024 Feb 16;229(3):741–58. doi: 10.1007/s00429-023-02750-8 (PMC10978731; doi:10.1007/s00429-023-02750-8)
Supplement: Supplementary file 1 — Supplementary file1 (DOCX 60 KB) [file 429_2023_2750_MOESM1_ESM.docx]

**Supplementary Material**

**Supplementary Table 1**. Categories assigned to the 174 abstracts approaching sex/gender and submitted for a poster presentation at the OHBM 2022.

| **Title** | **Analytical category** | **Topic category** |
| --- | --- | --- |
| [A comparison of intracranial volume estimation methods and their associations with age](https://event.fourwaves.com/ohbm-2022/abstracts/85e80a12-027c-4cab-984b-d446c99ca0f9) | Sex/gender as a binary variable | Modeling and analysis methods |
| [A connectome biomarker for blood pressure: a predictive analysis in 31367 UK Biobank participants](https://event.fourwaves.com/ohbm-2022/abstracts/7cb5d368-9608-446a-ba81-251cb65bc9a0) | Sex/gender as a covariate | Neurophysiology |
| [A Critical Note on the Right Hemispheric Dominance of the Face Perception Network](https://event.fourwaves.com/ohbm-2022/abstracts/b2f720f9-3625-4d42-b3f7-3b8761e6f403) | Sex/gender as a binary variable | Cognitive, affective and behavioral neuroscience |
| [A high-resolution autoencoder for construction of interpretable brain MRI endophenotypes](https://event.fourwaves.com/ohbm-2022/abstracts/d0d154e2-c100-492c-b94c-8ce7e67819f3) | Sex/gender as a covariate | Modeling and analysis methods |
| [A novel graph matching-based metric of the human connectome varies with age and sex](https://event.fourwaves.com/ohbm-2022/abstracts/3747e541-7fe0-4c86-ba97-272580d9687e) | Sex/gender as a covariate | Modeling and analysis methods |
| [A unified GAN model to predict an individual's 3D brain MRI at older and younger ages](https://event.fourwaves.com/ohbm-2022/abstracts/085817cf-ebf0-43be-867a-d24d166f71a0) | Sex/gender as a covariate | Lifespan developmental |
| [Aberrant Striatal White Matter Connectivity and Microstructure in Obsessive-Compulsive Disorder](https://event.fourwaves.com/ohbm-2022/abstracts/fdf9b506-10b3-4322-a8c7-662fb1e71f51) | Sex/gender as a covariate | Psychiatric disorders |
| [Adjusting for Nuisance Variables in Neuroimaging Studies via Penalized Decomposition Using Residuals](https://event.fourwaves.com/ohbm-2022/abstracts/b91d3065-7389-45aa-ab8c-fdb410ba3e16) | Sex/gender as a covariate | Modeling and analysis methods |
| [Adolescents’ self-concept is associated with functional connectivity in the anterior cingulate](https://event.fourwaves.com/ohbm-2022/abstracts/aca34435-6936-486d-aaeb-650b55bd1023) | Sex/gender as a binary variable | Cognitive, affective and behavioral neuroscience |
| [Advanced brain age in schizophrenia: a worldwide ENIGMA-Schizophrenia study.](https://event.fourwaves.com/ohbm-2022/abstracts/f90f3e2d-baac-4960-bab9-06c7c8dbb6e2) | Sex/gender as a covariate | Psychiatric disorders |
| [Advanced Diffusion-Weighted MRI Metrics are Associated with Pubertal Development in over 6,000 Youth](https://event.fourwaves.com/ohbm-2022/abstracts/8ab04af3-a3fc-45ca-9c92-be7160a7639d) | Sex/gender as a binary variable | Lifespan developmental |
| [Aging Effect on Caudate Function Differs Between Women and Men in Mild Cognitive Impairment](https://event.fourwaves.com/ohbm-2022/abstracts/c47005ae-ef36-4408-abb7-d7f3ec665512) | Sex/gender as a binary variable | Neurological and neurodegenerative disorders |
| [Alterations in whole-brain network connectivity in adults with a history of child abuse](https://event.fourwaves.com/ohbm-2022/abstracts/8c7d3eea-0afa-407d-aef0-d2d29530cbf2) | Sex/gender as a covariate | Psychiatric disorders |
| [Altered Brain Signal Complexity in fMRI Improves AD Classification in Deep Learning Model](https://event.fourwaves.com/ohbm-2022/abstracts/1fdc7f73-6586-43d0-a417-8b4b3541b6e4) | Sex/gender as a covariate | Modeling and analysis methods |
| [Altered correlation of simultaneously recorded EEG-fMRI connectomes in temporal lobe epilepsy](https://event.fourwaves.com/ohbm-2022/abstracts/5193fc2c-524a-48e1-be7c-81d5c42b63f6) | Sex/gender as a covariate | Neurological and neurodegenerative disorders |
| [Altered Hypothalamic Structure In Trigeminal Neuralgia](https://event.fourwaves.com/ohbm-2022/abstracts/c932f3e1-3147-4400-a620-c130886bf003) | Sex/gender as a binary variable | Neurological and neurodegenerative disorders |
| [Amygdala-Insula Resting-State Functional Connectivity Predicts Substance Use in Adolescents](https://event.fourwaves.com/ohbm-2022/abstracts/360e068e-a352-4e96-bff9-70a0821633bf) | Sex/gender as a covariate | Psychiatric disorders |
| [An Integrated Neurogenetic-Behavioral Signature Predicts Widespread Pain Development in Children](https://event.fourwaves.com/ohbm-2022/abstracts/7beb4202-0c1c-41d0-bb77-c808b6368c87) | Sex/gender as a covariate | Neurophysiology |
| [APOE ɛ2 vs APOE ɛ4 dosage shows sex-specific links with hippocampus-default network co-variation](https://event.fourwaves.com/ohbm-2022/abstracts/be58e179-f758-4ca1-9380-bd8903f29708) | Sex/gender as a binary variable | Neurological and neurodegenerative disorders |
| [Associations between brain volumes and future general cognitive ability in infancy and childhood](https://event.fourwaves.com/ohbm-2022/abstracts/1fc4066d-d640-427a-ae95-b231ca0996ee) | Sex/gender as a covariate | Lifespan developmental |
| [Associations of depression and regional brain structure across the adult lifespan: pooled analyses of 8 population-based and 2 clinical cohort studies in the European Lifebrain consortium](https://event.fourwaves.com/ohbm-2022/abstracts/1ececa76-3f07-40d8-abe3-77208083e902) | Sex/gender as a binary variable | Psychiatric disorders |
| [Associations of negative symptom scores with functional connectivity across resting state datasets](https://event.fourwaves.com/ohbm-2022/abstracts/ddc41ecb-5e6b-49ef-81a8-42edc0340adf) | Sex/gender as a covariate | Psychiatric disorders |
| [Automatically segmented olfactory bulb volume as a quantitative proxy of olfactory function](https://event.fourwaves.com/ohbm-2022/abstracts/8a151dc1-7584-488e-b644-7a52b232e833) | Sex/gender as a covariate | Neurophysiology |
| [BMI and Gender effects on association between BMI and resting state functional connectivity in reward network](https://event.fourwaves.com/ohbm-2022/abstracts/5655cbcb-3010-4b2d-9623-3393f0c3e766) | Sex/gender as a binary variable | Cognitive, affective and behavioral neuroscience |
| [Brain atrophy in REM sleep behavior disorder is shaped by gene expression and connectivity](https://event.fourwaves.com/ohbm-2022/abstracts/16fa070f-354b-41f0-aa62-f190d336f682) | Sex/gender as a covariate | Neurological and neurodegenerative disorders |
| [Brain morphological variability of the racial identity using HCP data](https://event.fourwaves.com/ohbm-2022/abstracts/55e75183-8a49-4bb4-b77d-117dc1aa066f) | Sex/gender as a covariate | Brain anatomy |
| [Brain structural sex differences in autism spectrum disorders: a systematic review](https://event.fourwaves.com/ohbm-2022/abstracts/3d448248-0f85-4c15-ae09-74ceec6aeff0) | Sex/gender as a binary variable | Neurodevelopmental and disruptive behavior disorders |
| [Cerebellar gradient of volume reduction in Fetal Alcohol Syndrome: toward a neuroanatomical marker?](https://event.fourwaves.com/ohbm-2022/abstracts/21209db3-d16c-4b42-8921-dcde7a1cf285) | Sex/gender as a covariate | Neurodevelopmental and disruptive behavior disorders |
| [Cerebral Perfusion Underpinnings of Functional Connectivity Networks in Temporal Lobe Epilepsy](https://event.fourwaves.com/ohbm-2022/abstracts/b850cbce-372c-475c-a24a-fba5a39a1b92) | Sex/gender as a covariate | Neurological and neurodegenerative disorders |
| [Changes in neurochemistry across early-middle childhood](https://event.fourwaves.com/ohbm-2022/abstracts/ac543f9f-1a7e-4036-a553-ee026774cffe) | Sex/gender as a binary variable | Lifespan developmental |
| [Charting Age and Sex Effects on White Matter Microstructure in 34,423 Adults Using Advanced Metrics](https://event.fourwaves.com/ohbm-2022/abstracts/120ea5c7-ec52-4b49-9b4c-bffe1c9b59c3) | Sex/gender as a binary variable | Lifespan developmental |
| [Charting human subcortical maturation across the adult lifespan with in vivo 7 T MRI](https://event.fourwaves.com/ohbm-2022/abstracts/e3c6a70a-0b53-47e3-86d4-efd7cbe8be7a) | Sex/gender as a binary variable | Lifespan developmental |
| [Cognitive correlates of age/sex differences in functional connectivity after traumatic brain injury](https://event.fourwaves.com/ohbm-2022/abstracts/f9de2ce1-3a49-4a32-9516-0b480e1cde3c) | Sex/gender as a binary variable | Neurological and neurodegenerative disorders |
| [Confounds in Neuroimaging: A Clear Case of Sex as a Confound in Brain Prediction](https://event.fourwaves.com/ohbm-2022/abstracts/eb7ca59c-83fd-41b8-ba86-9a0fc2031557) | Sex/gender as a binary variable | Modeling and analysis methods |
| [Consistency in sex classification analyses in brain functional organization across big data sets](https://event.fourwaves.com/ohbm-2022/abstracts/fa0b8d4f-39ad-46c6-ab03-bf7dd1ca58d6) | Sex/gender as a binary variable | Modeling and analysis methods |
| [Constancy of sex differences during dynamic adolescent development of morphometric networks](https://event.fourwaves.com/ohbm-2022/abstracts/cb0c9af9-8c43-4485-b0cc-4799e464c28f) | Sex/gender as a binary variable | Lifespan developmental |
| [Contralesional White Matter May Support Language Processing in Perinatal Stroke](https://event.fourwaves.com/ohbm-2022/abstracts/03cd7441-da32-4a35-8083-9250cb29c9f7) | Sex/gender as a covariate | Neurological and neurodegenerative disorders |
| [Control for brain volume in biological sex classification of cisgender and transgender individuals](https://event.fourwaves.com/ohbm-2022/abstracts/52cce116-aa39-4fc3-8d8f-9ccd2f3a1852) | Sex/gender with additional social information | Modeling and analysis methods |
| [CORRELATION BETWEEN HIPPOCAMPUS VOLUME AND SYMPTOM SEVERITYIN PATIENTS WITH OCD: AN MRI STUDY](https://event.fourwaves.com/ohbm-2022/abstracts/5a9ff033-77f9-453a-97d5-d74965266436) | Sex/gender as a covariate | Psychiatric disorders |
| [Decode age-specific developing patterns in brain functional connectivity during adolescence](https://event.fourwaves.com/ohbm-2022/abstracts/1dab68c3-f115-45f5-9b6b-ca9d47822bed) | Sex/gender as a binary variable | Lifespan developmental |
| [Deep learning identifies robust sex differences in functional brain organization and their dissociable links to clinical symptoms in autism](https://event.fourwaves.com/ohbm-2022/abstracts/95d31ffe-b2dc-4d95-9dba-fd5e261a9221) | Sex/gender as a binary variable | Neurodevelopmental and disruptive behavior disorders |
| [DEEP LEARNING PREDICTION OF GENDER FROM LONGITUDINAL STRUCTURAL MRI DATA IN THE ABCD STUDY](https://event.fourwaves.com/ohbm-2022/abstracts/3423c093-37b3-4aa9-872c-c1e353a10e6d) | Sex/gender as a binary variable | Modeling and analysis methods |
| [Default mode connectivity tracks Alzheimer’s biomarkers in a racially diverse middle-aged cohort](https://event.fourwaves.com/ohbm-2022/abstracts/5bddc996-831a-441a-bed7-36a25e02c04a) | Sex/gender as a covariate | Neurological and neurodegenerative disorders |
| [Denoising Approach Affects Diagnostic Differences in Brain Connectivity across Alzheimer’s Continuum](https://event.fourwaves.com/ohbm-2022/abstracts/9588a78c-cf95-479f-9b4e-54336be1d8ed) | Sex/gender as a covariate | Modeling and analysis methods |
| [Diagnosis and Sex in Autism Spectrum Disorder Explored using Phenomics, Genetics, and Neuroimaging](https://event.fourwaves.com/ohbm-2022/abstracts/94a5f3f5-bc7b-43bc-86b6-cd55441aa12a) | Sex/gender as a binary variable | Neurodevelopmental and disruptive behavior disorders |
| [Differences in Retinotopic Organization of Early Visual Areas after Temporary Congenital Blindness](https://event.fourwaves.com/ohbm-2022/abstracts/a99e53b7-7659-443c-a232-078c4129953b) | Sex/gender as a covariate | Neurophysiology |
| [Direct linkage detection with Multimodal IVA fusion: uncovering joint biomarkers in large studies](https://event.fourwaves.com/ohbm-2022/abstracts/a11209d2-f611-4423-936e-91808fc52e0b) | Sex/gender as a covariate | Modeling and analysis methods |
| [Distinct developmental changes in youth with ADHD: A longitudinal voxel-based morphometry study](https://event.fourwaves.com/ohbm-2022/abstracts/9f90d5ca-8a99-4730-8b78-e60ec4732291) | Sex/gender as a covariate | Neurodevelopmental and disruptive behavior disorders |
| [dTGA adults show persistent brain abnormalities as well as decreased global and regional volumes](https://event.fourwaves.com/ohbm-2022/abstracts/32fc79c3-a89b-4559-b0c7-e669cbcda6f5) | Sex/gender as a covariate | Neurological and neurodegenerative disorders |
| [Dynamic Changes in the Central Autonomic Network of Patients with Anorexia Nervosa](https://event.fourwaves.com/ohbm-2022/abstracts/45447dc0-abc4-4975-a53c-34486c94583a) | Sex/gender as a covariate | Psychiatric disorders |
| [Dynamics of Resting state connectivity predict anxiety in a transdiagnostic pediatric sample](https://event.fourwaves.com/ohbm-2022/abstracts/7da7302e-b74a-45cc-b614-b375a57e167a) | Sex/gender as a binary variable | Psychiatric disorders |
| [Effects of Age, Sex, and Handedness on Lateralization of Resting State Networks](https://event.fourwaves.com/ohbm-2022/abstracts/3351fe90-dd0b-4ea4-9495-2f53f659b7a9) | Sex/gender as a binary variable | Lifespan developmental |
| [Estimate gender-specific age-related glymphatic function changes with resting-state functional MRI](https://event.fourwaves.com/ohbm-2022/abstracts/b2e69aca-65cd-40b2-9fb6-ce67867b559b) | Sex/gender as a binary variable | Lifespan developmental |
| [Examining the interaction between prenatal stress and polygenic risk for ADHD on brain growth](https://event.fourwaves.com/ohbm-2022/abstracts/cea7317b-c949-4d8c-8efe-9a88cf0bffec) | Sex/gender as a binary variable | Neurodevelopmental and disruptive behavior disorders |
| [Exploring Demographic Effects on Regional Brain Volumes in a Large-Scale MRI Study](https://event.fourwaves.com/ohbm-2022/abstracts/853611a5-05cb-415e-8cce-4181f58db77d) | Sex/gender as a binary variable | Modeling and analysis methods |
| [Exploring white matter in treatment-resistant depression using diffusion MRI and free-water imaging](https://event.fourwaves.com/ohbm-2022/abstracts/400ce349-ef18-4785-a468-6d487a6ec363) | Sex/gender as a covariate | Psychiatric disorders |
| [Fetal testosterone is associated with sex differences in brain structure](https://event.fourwaves.com/ohbm-2022/abstracts/57bc44c6-db5f-432f-8346-574f82afe6af) | Sex/gender with additional biological information | Brain anatomy |
| [Functional Connectivity in Adolescents Assigned Female at Birth who Experience Gender Dysphoria](https://event.fourwaves.com/ohbm-2022/abstracts/64b89521-e30d-460b-8641-5a12d62bdeb6) | Sex/gender with additional social information | Cognitive, affective and behavioral neuroscience |
| [Gender incongruence and autistic traits: cerebral and behavioural underpinnings](https://event.fourwaves.com/ohbm-2022/abstracts/84016b86-1cd1-46bd-89b5-4d33ef6bf777) | Sex/gender as a binary variable | Neurodevelopmental and disruptive behavior disorders |
| [Glutamate Concentration of Dorsal Cingulate Modulates the Connectivity within the Salience Network](https://event.fourwaves.com/ohbm-2022/abstracts/2cf93f94-a9f7-4147-85e1-ae57dea68980) | Sex/gender as a covariate | Other |
| [Grey matter morphometry and MRI data-driven classification of premenstrual dysphoric disorder](https://event.fourwaves.com/ohbm-2022/abstracts/fb6a6e2f-6a11-41ca-b207-d4a704afb200) | Sex/gender with additional biological information | Psychiatric disorders |
| [Hallucination Severity in First-Episode Psychosis and STG-Thalamic Functional Connectivity in fMRI](https://event.fourwaves.com/ohbm-2022/abstracts/a3cba26f-0716-42f0-a687-dcb531098153) | Sex/gender as a covariate | Psychiatric disorders |
| [Harmonized TBSS Reveals Structural Abnormalities in White Matter Tracts in Patients with FCD](https://event.fourwaves.com/ohbm-2022/abstracts/b1228407-1e91-4148-9c63-008dac599d8c) | Sex/gender as a covariate | Neurological and neurodegenerative disorders |
| [High Oxford Happiness Scores Associated with Increased Cerebral Blood Flow in Reward Circuit](https://event.fourwaves.com/ohbm-2022/abstracts/1fc29bee-e210-4c36-a150-814de13f2529) | Sex/gender as a covariate | Cognitive, affective and behavioral neuroscience |
| [High Pulse Pressure is Associated with Changes in Brain Structure and Function in Healthy Volunteers](https://event.fourwaves.com/ohbm-2022/abstracts/5d10577b-2d0c-4863-8143-252eaf73cc12) | Sex/gender as a covariate | Neurophysiology |
| [How does the pregnant brain regulate emotions? – An fMRI study.](https://event.fourwaves.com/ohbm-2022/abstracts/62ba1910-7ef2-4eff-9943-11d0d539ab87) | Sex/gender with additional biological information | Cognitive, affective and behavioral neuroscience |
| [Human Brain Development: a cross-sectional and longitudinal study integrating multiple neuromorphological features](https://event.fourwaves.com/ohbm-2022/abstracts/0cdbf35c-0bd1-440d-a669-eb7df256f0db) | Sex/gender as a binary variable | Lifespan developmental |
| [Human intracortical structure differs between sexes and is modulated by the menstrual cycle](https://event.fourwaves.com/ohbm-2022/abstracts/e3d76a9e-46b0-4d72-9279-44faa4ddfe88) | Sex/gender with additional biological information | Brain anatomy |
| [Hyper segregation of functional brain networks and accelerated age-related segregation loss in mid-life cognitively healthy APOE-ɛ4 carriers](https://event.fourwaves.com/ohbm-2022/abstracts/d0508c3f-be74-4cd7-9289-e6113216097d) | Sex/gender as a covariate | Neurological and neurodegenerative disorders |
| [Identifying sources of bias when using two available tools for quantifying white matter lesions: BIANCA and LST](https://event.fourwaves.com/ohbm-2022/abstracts/69a67c60-bc5d-4a81-9b25-973cd1b63d50) | Sex/gender as a binary variable | Modeling and analysis methods |
| [Impact of sex on trajectories of cortical thinning in logopenic variant primary progressive aphasia](https://event.fourwaves.com/ohbm-2022/abstracts/22e180ef-9e87-455b-a819-b40f1f1ddb4e) | Sex/gender as a binary variable | Neurological and neurodegenerative disorders |
| [Impulse control correlates with brain functional connectivity among typically developing adolescents](https://event.fourwaves.com/ohbm-2022/abstracts/99a9561f-214f-438c-b7db-9a8fb9dafea9) | Sex/gender as a covariate | Cognitive, affective and behavioral neuroscience |
| [Indices of cortical asymmetry in Alzheimer's Disease and Frontotemporal Dementia](https://event.fourwaves.com/ohbm-2022/abstracts/0d60ed6e-69db-4f54-96de-e482d30def78) | Sex/gender as a covariate | Neurological and neurodegenerative disorders |
| [Individual Variability in Brain Self-regulation by Neurofeedback Associated with Treatment Effects](https://event.fourwaves.com/ohbm-2022/abstracts/89b94c74-e3d1-4f04-bd0d-bed020ea1d70) | Sex/gender as a covariate | Psychiatric disorders |
| [Individual variability in structural brain development with respect to sex and puberty](https://event.fourwaves.com/ohbm-2022/abstracts/4d32438b-266e-402a-9b4f-4a04551d41ec) | Sex/gender as a binary variable | Lifespan developmental |
| [Influence of literacy status on hippocampal subfield structure in older adults](https://event.fourwaves.com/ohbm-2022/abstracts/0f1695bd-e644-4838-bf02-cdb9fb89e92a) | Sex/gender as a binary variable | Lifespan developmental |
| [Inhibitory Response Neurotypes Differ in Cognition, Psychopathology, & Default Mode Network Cohesion](https://event.fourwaves.com/ohbm-2022/abstracts/a3605d56-5016-4e2f-9b25-7056d6650bdc) | Sex/gender as a covariate | Cognitive, affective and behavioral neuroscience |
| [Interactive effects of HIV infection and chronic cannabis use on hippocampal rsFC](https://event.fourwaves.com/ohbm-2022/abstracts/6d5dcb26-07b3-4805-a08d-e92d7ed2971e) | Sex/gender as a covariate | Other |
| [Interpreting DTI Metrics for Charting White Matter Development Using Multiple Diffusion Models](https://event.fourwaves.com/ohbm-2022/abstracts/dc14c841-45ca-4ee8-8e57-5b092ba33405) | Sex/gender as a covariate | Modeling and analysis methods |
| [Intracranial Volume Correction Differentially Biases Brain-Behavior Predictions Across Populations](https://event.fourwaves.com/ohbm-2022/abstracts/0a631f20-97c9-4943-b8e7-ca12e7080f5d) | Sex/gender as a binary variable | Modeling and analysis methods |
| [Investigating seasonal influences on brain GABA and glutamate levels](https://event.fourwaves.com/ohbm-2022/abstracts/b75f260e-cdb4-410a-abc2-3920a31d744e) | Sex/gender as a covariate | Other |
| [Investigating the Neural Correlates of Gender Stereotype Threat](https://event.fourwaves.com/ohbm-2022/abstracts/89919fee-7bf7-4ab6-aca6-bce04c76b9be) | Sex/gender with additional social information | Cognitive, affective and behavioral neuroscience |
| [Large-scale predictive modeling of confound-prone motor targets with neuroimaging features from UKB](https://event.fourwaves.com/ohbm-2022/abstracts/ca65e6a0-8bfe-4b6f-8669-1e5298291e6f) | Sex/gender as a binary variable | Modeling and analysis methods |
| [Latent space representation of task-fMRI with semi-supervised Autoencoder](https://event.fourwaves.com/ohbm-2022/abstracts/1a3c6241-3e6d-432c-8af3-cfa500ceda19) | Sex/gender as a binary variable | Modeling and analysis methods |
| [Linked development of diffusion, NODDI, and functional connectivity measures through early childhood](https://event.fourwaves.com/ohbm-2022/abstracts/efc2ec0c-9f68-4f33-ae0f-c95109547935) | Sex/gender as a binary variable | Lifespan developmental |
| [Linking morphometric patterns of the psychosis spectrum to cognition and functional gradients](https://event.fourwaves.com/ohbm-2022/abstracts/6cfd93e8-f7b6-42c0-8175-0963057eb683) | Sex/gender as a binary variable | Psychiatric disorders |
| [Localized sex differences in anatomical brain-age estimates](https://event.fourwaves.com/ohbm-2022/abstracts/14786f21-425a-4d24-9125-c1927394bcd8) | Sex/gender as a binary variable | Lifespan developmental |
| [Long COVID: Aberrant connectivity of salience network in critically-ill patients](https://event.fourwaves.com/ohbm-2022/abstracts/af6a1342-a509-4340-b06c-dc7235a673d2) | Sex/gender as a covariate | Other |
| [Longitudinal analyses of cortical structure in the ABCD Study: Sex-by-age interactions](https://event.fourwaves.com/ohbm-2022/abstracts/da7c29c6-0dc2-4bf1-985c-9696f544efaa) | Sex/gender as a binary variable | Lifespan developmental |
| [Long-term changes of cerebellar structure after premature birth](https://event.fourwaves.com/ohbm-2022/abstracts/5855452a-f75f-441b-a6dd-2d6987c93979) | Sex/gender as a covariate | Brain anatomy |
| [Long-term endurance training induces cerebral perfusion changes in young Adults](https://event.fourwaves.com/ohbm-2022/abstracts/4d0f710f-13ab-49d6-827b-b58e313c116b) | Sex/gender as a binary variable | Neurophysiology |
| [Magnetic susceptibility and mental health symptoms in children with and without prenatal alcohol exposure](https://event.fourwaves.com/ohbm-2022/abstracts/1b7d9768-f2ed-4da9-970d-2c5e592a7e50) | Sex/gender as a covariate | Neurodevelopmental and disruptive behavior disorders |
| [Mapping brain structural and neuroreceptor correlates in Lewy Body visual hallucinations](https://event.fourwaves.com/ohbm-2022/abstracts/221c5a4a-b050-494f-a8e6-c337c4a8ae21) | Sex/gender as a covariate | Neurological and neurodegenerative disorders |
| [Mapping Frontal Corticostriatal Circuits in ADHD, Sex, Age, and Inhibitory Control](https://event.fourwaves.com/ohbm-2022/abstracts/dbcfa2f0-1845-41bf-a925-4d3af3bb860d) | Sex/gender as a binary variable | Neurodevelopmental and disruptive behavior disorders |
| [Mixing BrainAGE with omics approaches: Employing sPLS regression on UK Biobank data](https://event.fourwaves.com/ohbm-2022/abstracts/7b44e33e-9231-4fa1-8907-84203c1b6311) | Sex/gender as a binary variable | Modeling and analysis methods |
| [MRI functional connectivity sex differences of nicotine smoking](https://event.fourwaves.com/ohbm-2022/abstracts/b898e2b0-c770-42a2-a417-be6d664ac00f) | Sex/gender as a binary variable | Cognitive, affective and behavioral neuroscience |
| [Multilayer connectivity captures sex differences in functional brain connectivity throughout aging](https://event.fourwaves.com/ohbm-2022/abstracts/7d4c7fa4-8d0a-4da8-adc6-1e4caa24075d) | Sex/gender as a binary variable | Lifespan developmental |
| [Multimodal prediction of cognitive performance differences in older age](https://event.fourwaves.com/ohbm-2022/abstracts/a6ada68e-d9a3-4610-9074-4c04e84eb10e) | Sex/gender as a covariate | Lifespan developmental |
| [Neighborhood socioeconomic disadvantage and the neurobiology of uncertainty in traumatically injured](https://event.fourwaves.com/ohbm-2022/abstracts/a9c07f8f-0cbf-4f20-bc19-3f71a9390ef5) | Sex/gender as a covariate | Cognitive, affective and behavioral neuroscience |
| [Neonatal white matter microstructure correlates of 18-month neurodevelopmental outcomes](https://event.fourwaves.com/ohbm-2022/abstracts/e183bd05-1875-4bfa-906b-debf4cfc8c65) | Sex/gender as a covariate | Brain anatomy |
| [Neural and sociocultural mediators of gender differences in pain](https://event.fourwaves.com/ohbm-2022/abstracts/689e507f-a971-443c-b564-d9fb92815f6f) | Sex/gender as a binary variable | Neurophysiology |
| [Neural processing of benevolent, tickling and taunting laughter in disruptive behavior disorders](https://event.fourwaves.com/ohbm-2022/abstracts/49504ac4-4298-42dd-af32-c403c683d323) | Sex/gender as a covariate | Neurodevelopmental and disruptive behavior disorders |
| [Neural underpinnings of individual differences in emotion regulation](https://event.fourwaves.com/ohbm-2022/abstracts/32d7d633-ec4a-4247-bb33-832fb04f3954) | Sex/gender as a binary variable | Cognitive, affective and behavioral neuroscience |
| [Neurobiological and psychosocial factors in alcohol-use disorder](https://event.fourwaves.com/ohbm-2022/abstracts/61b1a611-aa45-4134-a35c-55670efa8f83) | Sex/gender as a binary variable | Psychiatric disorders |
| [Neuroimaging VMAT2 in Parkinson’s Disease with rapid eye movement sleep behaviour disorder](https://event.fourwaves.com/ohbm-2022/abstracts/df5d36b0-50e4-46ae-ba97-3bf3ce080fdd) | Sex/gender as a covariate | Neurological and neurodegenerative disorders |
| [Neurometabolite correlates with personality and stress in healthy emerging adults: sex differences](https://event.fourwaves.com/ohbm-2022/abstracts/23bd2942-236d-4fe3-8a0d-0bdad0c0bbaf) | Sex/gender as a binary variable | Cognitive, affective and behavioral neuroscience |
| [Neuroplasticity is associated with altered whole brain connectivity in prodromal Alzheimer's Disease](https://event.fourwaves.com/ohbm-2022/abstracts/f70d56f6-be9a-450a-9a37-ec62c706b898) | Sex/gender as a covariate | Neurological and neurodegenerative disorders |
| [Normative modeling of sex differences in neurodevelopment of autism and ADHD](https://event.fourwaves.com/ohbm-2022/abstracts/264b63c7-dae1-4e6d-9310-7edf3d7cfd60) | Sex/gender as a binary variable | Neurodevelopmental and disruptive behavior disorders |
| [Parenthood affects age-related alterations of whole-brain controllability](https://event.fourwaves.com/ohbm-2022/abstracts/dbc5d0ae-3c42-40dd-ba4e-3ce285b612be) | Sex/gender as a binary variable | Lifespan developmental |
| [Paternal Care Affects Left Superior Parietal Lobule Gray Matter Volume in Healthy Adult Offspring](https://event.fourwaves.com/ohbm-2022/abstracts/b458fcba-4a9c-4dee-84c0-234adee11f18) | Sex/gender as a covariate | Cognitive, affective and behavioral neuroscience |
| [PHOTONAI-Graph - A Python Toolbox for Graph Machine Learning on Neuroimaging Data](https://event.fourwaves.com/ohbm-2022/abstracts/a9166226-f540-4166-a1ca-8c0dadd4f11a) | Sex/gender as a covariate | Modeling and analysis methods |
| [Polygenic risk for depression moderates association between amygdala connectivity and internalizing](https://event.fourwaves.com/ohbm-2022/abstracts/955e77ac-7830-4c7c-a8ea-3c9a7497cbec) | Sex/gender as a binary variable | Psychiatric disorders |
| [Predicting age-related cortical changes: Can an online battery match in-person cognitive testing?](https://event.fourwaves.com/ohbm-2022/abstracts/d9f33cd2-f68c-43aa-ad7c-22d32b27ff61) | Sex/gender as a binary variable | Modeling and analysis methods |
| [Predicting influence of X chromosome on neuroanatomy and behavior using support vector regression](https://event.fourwaves.com/ohbm-2022/abstracts/c262f217-7d1a-4711-9282-6a3659e868d6) | Sex/gender with additional biological information | Brain anatomy |
| [Predicting Transdiagnostic Social Impairments in Childhood using Connectome-based Predictive Modelin](https://event.fourwaves.com/ohbm-2022/abstracts/a9045cfc-03de-4f8d-8dc3-659fa22fe1fc) | Sex/gender as a covariate | Neurodevelopmental and disruptive behavior disorders |
| [PREDICTION OF PERFORMANCE ON THE CLOCK DRAWING TEST FROM FRACTIONAL ANISOTROPY](https://event.fourwaves.com/ohbm-2022/abstracts/acc27b18-3880-44d3-bed8-17ccf61a568e) | Sex/gender as a covariate | Cognitive, affective and behavioral neuroscience |
| [Prediction of subject biological sex using blood delay map and correlation strength map features](https://event.fourwaves.com/ohbm-2022/abstracts/9dd47c3a-56a0-4dc3-93ec-85a3554a6f61) | Sex/gender as a covariate | Modeling and analysis methods |
| [Predictive Modeling of Delayed Reward Discounting in the Human Connectome Project](https://event.fourwaves.com/ohbm-2022/abstracts/c851c5ae-f023-4c14-bf8a-311b1d381c65) | Sex/gender as a covariate | Modeling and analysis methods |
| [Predictive modeling of pain sensitivity using cortical thickness](https://event.fourwaves.com/ohbm-2022/abstracts/885b226f-13f7-415c-b02f-372b376d0486) | Sex/gender as a covariate | Modeling and analysis methods |
| [Predictors of adolescent drug use: Working memory-related PCC deactivation and cognitive failures](https://event.fourwaves.com/ohbm-2022/abstracts/ea6f9b33-1ba1-4c68-a12f-166b7fc31fb0) | Sex/gender as a covariate | Cognitive, affective and behavioral neuroscience |
| [Reconstructing atlas-specific connectomes from data previously processed with different atlases](https://event.fourwaves.com/ohbm-2022/abstracts/28a32887-aa13-46a1-8599-2a39d393bd58) | Sex/gender as a covariate | Modeling and analysis methods |
| [Regional effects of age and sex within APOE genotype on cerebral perfusion measures](https://event.fourwaves.com/ohbm-2022/abstracts/acb0395d-467b-4cdf-aab4-e3258ecace39) | Sex/gender as a binary variable | Neurological and neurodegenerative disorders |
| [Resting-state Connectivity may predict treatment outcomes in youths](https://event.fourwaves.com/ohbm-2022/abstracts/4937b7e4-34d5-456b-a76a-7d7869acfbba) | Sex/gender as a covariate | Cognitive, affective and behavioral neuroscience |
| [Revealing neuroanatomical heterogeneity of Alzheimer's disease using normative modelling](https://event.fourwaves.com/ohbm-2022/abstracts/99e2b9a5-0b24-496b-869f-0d7d1c6cca88) | Sex/gender as a covariate | Neurological and neurodegenerative disorders |
| [Reward-related resting state functional connectivity in adolescent cannabis use](https://event.fourwaves.com/ohbm-2022/abstracts/e9147fe1-de7b-4c1a-a58f-ed4a0883801d) | Sex/gender as a covariate | Cognitive, affective and behavioral neuroscience |
| [Robust and Interpretable Deep Learning Methods for Discovering Biological Sex Differences](https://event.fourwaves.com/ohbm-2022/abstracts/ed8264ca-3df6-46f7-8e94-08de3d008fba) | Sex/gender as a binary variable | Modeling and analysis methods |
| [Schizophrenia influences response of the theory of mind network to socially awkward events](https://event.fourwaves.com/ohbm-2022/abstracts/0b4c7b34-c8a4-4659-b76e-06f21db30cb2) | Sex/gender as a covariate | Psychiatric disorders |
| [Schizophrenia polygenic risk scores negatively associated with white matter volumes in term neonates](https://event.fourwaves.com/ohbm-2022/abstracts/eaa267aa-1684-4926-9c11-b27e9f392201) | Sex/gender as a covariate | Psychiatric disorders |
| [Sex and age differences in resting state functional connectivity networks after mild traumatic brain](https://event.fourwaves.com/ohbm-2022/abstracts/7f10acf9-864f-4c7e-9adc-89317ef7a929) | Sex/gender as a binary variable | Neurological and neurodegenerative disorders |
| [Sex and gender effects on cortical thickness in 9- and 10-year-olds](https://event.fourwaves.com/ohbm-2022/abstracts/54d949b9-8d2f-4f0c-a6dd-7af197359027) | Sex/gender with additional social information | Brain anatomy |
| [Sex differences in social anxiety: Neural temporal dynamics of acceptance emotion regulation](https://event.fourwaves.com/ohbm-2022/abstracts/57a012a0-eb6d-4282-9d62-1fd825b5de7a) | Sex/gender as a binary variable | Cognitive, affective and behavioral neuroscience |
| [Sex Hormones & Medial Temporal Lobe: 7T MRI Shows Volume Changes at Subregion Level over Menstrual Cycle](https://event.fourwaves.com/ohbm-2022/abstracts/9eb7274d-b62f-4ec7-9485-16968bfb9989) | Sex/gender with additional biological information | Brain anatomy |
| [Sex Moderates the Relationship between Functional Connectivity and Remission in Late-life Depression](https://event.fourwaves.com/ohbm-2022/abstracts/4afcc22a-0d8f-4253-8871-8260d09791d2) | Sex/gender as a binary variable | Psychiatric disorders |
| [Sex/gender-related aspects in brain functional connectivity during language processing](https://event.fourwaves.com/ohbm-2022/abstracts/1c952c63-793b-4764-a182-0846081bf87f) | Sex/gender with additional social information | Cognitive, affective and behavioral neuroscience |
| [Sex-specific association of amygdala activity with memory performance](https://event.fourwaves.com/ohbm-2022/abstracts/84d02053-1fb0-42e4-8f84-73d0bc0a6eb1) | Sex/gender as a binary variable | Cognitive, affective and behavioral neuroscience |
| [Sex-Specific Effects of Prenatal Famine on Resting-State Functional Connectivity in the Human Brain](https://event.fourwaves.com/ohbm-2022/abstracts/77521009-ca81-4d06-9710-cc5bdc3d96ac) | Sex/gender as a binary variable | Neurodevelopmental and disruptive behavior disorders |
| [Sex-Specific Impacts of Stress During Childhood and Adulthood in The Brain: A UK Biobank Study](https://event.fourwaves.com/ohbm-2022/abstracts/27a64f54-7750-40d7-8291-05eb770d913e) | Sex/gender as a binary variable | Lifespan developmental |
| [Social-Affective Resting State Connectivity across Start and Termination of Oral Contraceptive Use](https://event.fourwaves.com/ohbm-2022/abstracts/5e7097fe-4413-40c8-8d6a-40dddb4a557e) | Sex/gender with additional biological information | Cognitive, affective and behavioral neuroscience |
| [Specific neurolinguistic patterns in a gender-diverse population. A neurofeminist approach.](https://event.fourwaves.com/ohbm-2022/abstracts/a50f684b-d9db-4460-b009-d675e1874fb1) | Sex/gender with additional social information | Cognitive, affective and behavioral neuroscience |
| [Structural Brain Development Across Early Childhood in Reading and Language Regions](https://event.fourwaves.com/ohbm-2022/abstracts/ef536499-251f-412c-9b75-f83a8633d3ea) | Sex/gender as a covariate | Cognitive, affective and behavioral neuroscience |
| [Structural Connectivity in Acute Pediatric TBI - a Graph Theory Perspective](https://event.fourwaves.com/ohbm-2022/abstracts/ee816251-f513-4e7e-b843-216cc967f4d2) | Sex/gender as a covariate | Neurological and neurodegenerative disorders |
| [Structural Connectome of Reinforcement Learning Constructs using Multimodal Data Fusion](https://event.fourwaves.com/ohbm-2022/abstracts/5d2c1b31-808e-403b-9fee-b9d2eecc71e5) | Sex/gender as a covariate | Modeling and analysis methods |
| [Structural Co-Regression (SCoRe): A novel technique for subject-specific anatomical connectivity](https://event.fourwaves.com/ohbm-2022/abstracts/754650d6-b130-4e62-8e37-ff8d91ba3f98) | Sex/gender as a binary variable | Modeling and analysis methods |
| [Structural Neural Correlates of Driving Across the Aging Spectrum: A VR Driving Simulator MRI Study](https://event.fourwaves.com/ohbm-2022/abstracts/5377d7ef-4cfe-411d-a89d-95c138bd3911) | Sex/gender as a covariate | Lifespan developmental |
| [Subcortical Morphometric Differences and Heterogeneity in Autism Spectrum Disorder](https://event.fourwaves.com/ohbm-2022/abstracts/6b273c3a-c15c-4ca3-97ce-1ea75acb4edf) | Sex/gender as a binary variable | Neurodevelopmental and disruptive behavior disorders |
| [Task fMRI Prediction of Postsurgical Cognitive Outcomes in Temporal Lobe Epilepsy: A Meta-Analysis](https://event.fourwaves.com/ohbm-2022/abstracts/bad8769e-8d60-4530-abd7-5920f5cf8b38) | Sex/gender as a binary variable | Neurological and neurodegenerative disorders |
| [Task- versus rest-derived network models as predictors of task-based BOLD signal changes](https://event.fourwaves.com/ohbm-2022/abstracts/cb156309-37d1-450f-925d-af8296f65127) | Sex/gender as a covariate | Modeling and analysis methods |
| [The association of vascular cell adhesion molecule-1 with grey matter volume in depression](https://event.fourwaves.com/ohbm-2022/abstracts/9f11956d-2184-4974-8f20-c5e38b9644bc) | Sex/gender as a covariate | Psychiatric disorders |
| [The effects of time of day, age, and sex in resting-state fMRI](https://event.fourwaves.com/ohbm-2022/abstracts/7ec63466-d57d-4a74-bef5-9b69b70b5b49) | Sex/gender as a binary variable | Modeling and analysis methods |
| [The Fluidity of Age and Sex differences for Language based Functional Connectivity Networks](https://event.fourwaves.com/ohbm-2022/abstracts/8b10e402-889d-41cc-8d51-6e9c400c2a49) | Sex/gender as a binary variable | Cognitive, affective and behavioral neuroscience |
| [The Impact of Chronic Pain on Fornix White Matter Microstructure](https://event.fourwaves.com/ohbm-2022/abstracts/af5194a0-d1a7-433c-93c2-eda7c3e58d20) | Sex/gender as a covariate | Neurophysiology |
| [The Impact of Minority Stress on the Developing Brains of Gender Diverse Youth](https://event.fourwaves.com/ohbm-2022/abstracts/5bb18dd6-e6b7-4eb5-8f1f-017cc90236f2) | Sex/gender with additional social information | Cognitive, affective and behavioral neuroscience |
| [The influence of gender identity and sexual orientation on voice-gender perception](https://event.fourwaves.com/ohbm-2022/abstracts/03cbc8ee-fcda-486f-abc9-7f96cacd153d) | Sex/gender with additional social information | Cognitive, affective and behavioral neuroscience |
| [The long-term impact of activation of the mother’s immune system during pregnancy on brain morphology in late childhood](https://event.fourwaves.com/ohbm-2022/abstracts/9bdc0a22-9edb-4f26-ba29-c49dfec50ceb) | Sex/gender as a binary variable | Neurodevelopmental and disruptive behavior disorders |
| [The midpoint of cortical thinning in adolescence differs across individuals and brain regions](https://event.fourwaves.com/ohbm-2022/abstracts/534fee5d-d450-4c36-b94f-3a9f1d00b437) | Sex/gender as a binary variable | Lifespan developmental |
| [The olfactory networks elicited by sniffing in patients with congenital anosmia](https://event.fourwaves.com/ohbm-2022/abstracts/f3525543-4dbb-49ed-986d-7300899352e3) | Sex/gender as a covariate | Neurophysiology |
| [The relationship between the APOE ε4 allele, menopause & hippocampal atrophy in typical aging](https://event.fourwaves.com/ohbm-2022/abstracts/cedda200-2c0d-4eda-bfee-d62bd1154680) | Sex/gender as a binary variable | Lifespan developmental |
| [The role of sleep quality and grey matter volume in prediction of depressive symptoms severity](https://event.fourwaves.com/ohbm-2022/abstracts/c2534563-6683-42a1-b542-9b4e5edf18dd) | Sex/gender as a covariate | Psychiatric disorders |
| [Topological Data Analysis differentiates term and premature infants at term-equivalent age](https://event.fourwaves.com/ohbm-2022/abstracts/7c2690f2-7441-4b76-8716-8f1ef7a6000e) | Sex/gender as a covariate | Lifespan developmental |
| [Trans Identity Impacts Neural Correlates of Self-Other Referential Processing](https://event.fourwaves.com/ohbm-2022/abstracts/4ad0f6ab-445d-40e3-be6d-d26ab0aa57ac) | Sex/gender with additional social information | Cognitive, affective and behavioral neuroscience |
| [Trans-Diagnostic Structural Imaging in Psychosis: A Comparison Across Schizophrenia, Frontotemportal Dementia, and Alzheimer’s Disease](https://event.fourwaves.com/ohbm-2022/abstracts/407bea1f-68cc-46ad-8007-865b853e78f4) | Sex/gender as a covariate | Psychiatric disorders |
| [Typicality of adolescent brain responses to socioemotional stimuli reflects social phobia](https://event.fourwaves.com/ohbm-2022/abstracts/ea26b34f-6dd3-4594-aa3a-e2828d8e6e37) | Sex/gender as a covariate | Psychiatric disorders |
| [Understanding Gender-Specific Brain lateralization via Covariate-Dependent Machine Learning](https://event.fourwaves.com/ohbm-2022/abstracts/c30403b8-b30c-4fd3-ba2c-38531089941d) | Sex/gender as a binary variable | Modeling and analysis methods |
| [Unique and combined contribution of brain amyloid load and APOE4 Status on Brain Volume](https://event.fourwaves.com/ohbm-2022/abstracts/f4a1a023-65dd-4291-abe0-29fd8a26eef9) | Sex/gender as a covariate | Neurological and neurodegenerative disorders |
| [Used of MR R2* maps to study longitudinal Deferiprone effects on Substantia Nigra.](https://event.fourwaves.com/ohbm-2022/abstracts/2c8179d8-ba47-447d-b17e-44d4b9c373c1) | Sex/gender as a covariate | Neurological and neurodegenerative disorders |
| [Using sex and gender for grey matter and total intracranial volume prediction provides best results](https://event.fourwaves.com/ohbm-2022/abstracts/b5166a84-3cb2-4d01-870e-0b341b1a89aa) | Sex/gender with additional social information | Brain anatomy |
| [Variability in cognitive task performance, network segregation and externalizing psychopathology](https://event.fourwaves.com/ohbm-2022/abstracts/ad2290da-1a3b-488b-953f-121effd22487) | Sex/gender as a covariate | Cognitive, affective and behavioral neuroscience |
| [Volumetric differences in cerebellar subregions in PTSD: a PGC-ENIGMA PTSD workgroup study](https://event.fourwaves.com/ohbm-2022/abstracts/cafd1968-b6e5-42fd-88c4-6c0f7160c3f1) | Sex/gender as a covariate | Psychiatric disorders |
| [White Matter and the Two Factor Model of Negative Symptoms of Schizophrenia](https://event.fourwaves.com/ohbm-2022/abstracts/f719b29e-dc43-4d92-8ab9-595c50906381) | Sex/gender as a covariate | Psychiatric disorders |
| [White matter hyperintensity volume modifies the association between CSF vascular biomarkers and regional FDG-PET along the Alzheimer’s disease continuum](https://event.fourwaves.com/ohbm-2022/abstracts/e7505dd2-db0f-4f9b-b4af-f3a1019fc945) | Sex/gender as a binary variable | Neurological and neurodegenerative disorders |
| [White matter microstructural changes in speech impairments in Parkinson’s disease](https://event.fourwaves.com/ohbm-2022/abstracts/8da3913e-0f0c-4c50-96b9-812421cd894d) | Sex/gender as a covariate | Neurological and neurodegenerative disorders |
| [White Matter Microstructural Correlates of Spoken Discourse in Cerebrovascular Disease](https://event.fourwaves.com/ohbm-2022/abstracts/3643b53b-08bc-4c8b-808c-fad2444476fc) | Sex/gender as a binary variable | Neurological and neurodegenerative disorders |
| [White Matter Microstructure Indicates Sex/Gender Differences in Developmental Trajectory](https://event.fourwaves.com/ohbm-2022/abstracts/ffa6959b-b8da-4a03-986b-8e56fbf4b513) | Sex/gender with additional social information | Lifespan developmental |
| [White Matter Tract Length Differences with Age: Analysis of 18 Major Tracts Using TRACULA](https://event.fourwaves.com/ohbm-2022/abstracts/aec9fc31-a94b-4483-af88-a5b5305cbdd5) | Sex/gender as a binary variable | Lifespan developmental |
